# Applying the Consolidated Framework for Implementation Research to investigate factors of implementing alcohol screening and brief intervention among primary care physicians and nurses in Hong Kong, China: an exploratory sequential mixed-method study

**DOI:** 10.1186/s43058-024-00590-z

**Published:** 2024-05-06

**Authors:** Paul Shing-fong Chan, Yuan Fang, Yao Jie Xie, Martin Chi-sang Wong, Per Nilsen, Sau-fong Leung, Kin Cheung, Zixin Wang, Eng-kiong Yeoh

**Affiliations:** 1 Jockey Club School of Public Health and Primary Care, Faculty of Medicine, The Chinese University of Hong Kong, Hong Kong, China; 2 Department of Health and Physical Education, The Education University of Hong Kong, Hong Kong, China; 3 School of Nursing, The Hong Kong Polytechnic University, Hong Kong, China; 4 Division of Community Medicine, Department of Medical and Health Sciences, Linköping University, Linköping, Sweden

**Keywords:** Alcohol screening and brief intervention, Facilitators and barriers, Primary care settings, Consolidated Framework for Implementation Research, China, Mixed-method study

## Abstract

**Background:**

Alcohol screening and brief intervention (SBI) is an evidence-based intervention recommended by the World Health Organization. This study applied the Consolidated Framework for Implementation Research (CFIR) to understand facilitators and barriers of SBI implementation in primary care settings in Hong Kong, China.

**Methods:**

This was a sequential mixed-method study. In-depth interviews of 21 physicians and 20 nurses working in the primary care settings from the public and private sectors were first conducted to identify CFIR constructs that were relevant to SBI implementation in the Chinese context and potential factors not covered by the CFIR. A questionnaire was then developed based on the qualitative findings to investigate factors associated with SBI implementation among 282 physicians and 295 nurses.

**Results:**

The in-depth interviews identified 22 CFIR constructs that were facilitators or barriers of SBI implementation in Hong Kong. In addition, the stigmatization of alcohol dependence was a barrier and the belief that it was important for people to control the amount of alcohol intake in any situation was mentioned as a facilitator to implement SBI. In the survey, 22% of the participants implemented SBI in the past year. Factors associated with the SBI implementation echoed most of the qualitative findings. Among physicians and nurses in both sectors, they were more likely to implement SBI when perceiving stronger evidence supporting SBI, better knowledge and self-efficacy to implement SBI, more available resources, and clearer planning for SBI implementation in the clinics but less likely to do so when perceiving SBI implementation to be complicated and of higher cost, and drinking approved by the Chinese culture. Participants were more likely to implement SBI when perceiving SBI fit better with the existing practice and better leadership engagement in the public sector, but not in the private sector. Perceiving a stronger need and greater importance to implement SBI were associated with higher likelihood of SBI implementation among physicians, but not among nurses. Perceiving better organizational culture supporting SBI was positively associated with SBI implementation among nurses, but not among physicians.

**Conclusions:**

There was a significant gap between SBI evidence and its implementation. Some strategies to improve SBI implementation may be different between physicians and nurses and between those in the public and private sectors. The CFIR is a useful framework for understanding facilitators and barriers of SBI implementation in primary care settings.

**Supplementary Information:**

The online version contains supplementary material available at 10.1186/s43058-024-00590-z.

Contributions to the literature

- This was the first original study investigating the implementation of alcohol screening and brief intervention and its facilitators and barriers in the Chinese context.
- This was the first original study applying the Consolidated Framework for Implementation Research to understand SBI implementation in primary care settings.
- By integrating qualitative and quantitative methods and data, this study gained both breadth and depth of understanding of SBI implementation in the Chinese context.
- Some facilitators and barriers of SBI implementation were different between physicians and nurses, and between healthcare providers in the public and private sectors.

## Introduction

Worldwide, alcohol consumption is a leading cause of premature death and disability [1, 2]. A recent report showed that 9.3% of Chinese adults were heavy drinkers [3]. In Hong Kong, China, the study site, 70.5% of the people aged 18-64 years drank alcohol in the past year [4]. Among these drinkers, the prevalence of problematic alcohol consumption and binge drinking assessed with the Alcohol Use Disorders Identification Test (AUDIT) was 5% and 25%, respectively, in the last year [4]. Therefore, prevention and reduction of alcohol-related harm is a public health priority in Hong Kong.

Several meta-analyses have shown that Alcohol Screening and Brief Intervention (SBI), which refers to screening using standardized questions followed by brief and standardized advice or counseling for those exceeding certain drinking limits, could significantly reduce alcohol consumption among patients in primary care settings [5–8]. Cost-effectiveness analyses have also demonstrated that SBI led to significant savings on healthcare resources [9, 10]. Therefore, the World Health Organization (WHO) and other national health authorities strongly recommend SBI implementation in primary care settings [11–14]. However, there was a significant SBI research-practice gap, i.e. between evidence of SBI effectiveness and actual implementation of SBI in routine practice [15–19]. A recent systematic review showed that the practice rate was low among physicians in most countries on a regular basis (e.g., 17.2% in Finland, and 32.0% in the United States) [20]. The systematic review did not find any studies looking at SBI implementation in the Chinese context. A knowledge gap hence existed.

In Hong Kong, health services are provided by the public and private sectors [21]. In the public sector, primary care services are mainly provided by the General Out-Patient Clinic and Family Medicine Integrated/Specialist Clinic under the jurisdiction of the Hospital Authority. In the private sector, these services are provided by the General Out-Patient Clinic and Family Medicine Integrated/Specialist Clinic in private hospitals as well as clinics where physicians practice as solo or with partners/group practice [21]. The Hong Kong Department of Health produces a manual for primary care health professionals to conduct SBI and occasionally conducts SBI training workshops [22, 23]. In Hong Kong, the roles of physicians and nurses are different in primary care settings. The physicians are responsible for providing diagnoses/examinations, ordering tests, discussing test results, and prescribing medication/providing treatment, whilst the nurses are mainly responsible for recording patients’ medical information, carrying out health assessments, administering medication and treatment, and providing health education. As compared to the private sector, the job duties of physicians and nurses in the public sector are more hierarchical, more centrally assigned, and less flexible. However, there was no study investigating the implementation of SBI in Hong Kong.

The Consolidated Framework for Implementation Research (CFIR) (2009 version) is widely used to guide the systematic assessment of facilitators and barriers (determinants) that influence the implementation of many different evidence-based practices [24, 25]. The CFIR consists of five domains (i.e., types of determinants) that can be applied across the spectrum of implementation research [24, 25]: intervention characteristics (features of an intervention), inner setting (features of the organization), outer setting (features of external context or environment), characteristics of individuals (individuals involved in implementation), and implementation process (strategies or tactics) [24]. Each domain consists of a number of constructs. However, there is a dearth of studies applying the CFIR to study facilitators and barriers of SBI implementation in primary care settings [20]. Most published studies have focused on barriers and facilitators to SBI implementation under two of the five CFIR domains, characteristics of individuals (e.g., healthcare professionals’ knowledge, belief, and self-efficacy) and inner settings (e.g., available resources to deliver SBI) [20]. Potential factors of SBI implementation in the other three domains (intervention characteristics, outer settings, and implementation process) have been less studied [20].

To address the knowledge gaps, this study aimed to apply the CFIR to identify facilitators and barriers of SBI implementation among physicians and nurses in primary care settings in Hong Kong, China.

## Methods

### Study design

This study applied the 2009 version of CFIR [25] to identify facilitators and barriers of SBI implementation among physicians and nurses in primary care settings in Hong Kong, China. This study adopted an exploratory sequential mixed-method design. In-depth interviews of physicians and nurses were first conducted to identify CFIR constructs that were relevant to SBI implementation in the Chinese context and potential determinants not covered by the CFIR. This study was conducted between July 2021 and October 2022. As there was no study conducted to investigate the SBI implementation in Hong Kong, we first conducted the qualitative interview study to gather relevant information and identify the relevant CFIR constructs for SBI implementation in the local context and the results of the qualitative study aided the design of the quantitative survey. Ethics approval was obtained from the Survey and Behavioral Research Ethics Committee of the Chinese University of Hong Kong (SBRE-20-691).

### Qualitative study

Qualitative semi-structured face-to-face interviews were conducted with physicians and nurses working in primary care settings in Hong Kong. Interviews were conducted from July to December 2021.

#### Participants

Participants were full-time or part-time physicians and nurses working in the public sectors (General Out-Patient Clinic and Family Medicine Integrated/Specialist Clinic), or private sectors (General Out-Patient Clinic, Family Medicine Integrated/Specialist Clinic, clinics where physicians practice as solo or with partners/group practice). Physician interns or student nurses were excluded.

#### Recruitment and data collection

A quota sampling in line with the study inclusion criteria was adopted to ensure diversity among the participants. The population of subjects was divided into quotas based on physicians or nurses, gender (male or female), and type of clinics (public/private General Out-patient Clinic, public/private Family Medicine Integrated/Specialist Clinic, and private clinics as solo or group practice). Therefore, the predetermined sample size would be at least 2*2*6 = 24.

A face-to-face, semi-structured, individual in-depth interview with open-ended interview questions was conducted in Cantonese with eligible participants. Written informed consent was obtained prior to the interviews. Before the interview, participants were briefed about the interview procedure and they could seek clarifications. The interviews were conducted in quiet places with privacy to protect participants’ confidentiality and were audio-recorded with participants’ consent. Each interview lasted between 1.5 to 2 hours. A supermarket coupon valued at HK$150 (US$19.5) was given to each participant as a token of appreciation for their participation in the study.

#### Development of the interview guide

A panel consisting of three experts in health system research and public health was formed to develop the interview guide. We used open-ended questions which were adapted from the interview guide tool developed by the CFIR expert team [26] to collect facilitators and barriers and group them under relevant CFIR constructs. Some prompts were given to encourage the participants to think more and give sufficient information. There was flexibility for the participants to talk about new ideas brought up during the interview or elaborate on the points that were meaningful to them which would drive their answers deeper. The interview guide was pilot tested among three physicians and three nurses to assess the clarity and relevancy of the questions. Based on their comments, the panel revised and finalized the interview guide.

#### Data analysis

The interviews were transcribed verbatim after each interview and imported into Nvivo 12 for analysis. Transcripts were analyzed by directed content analysis. It is an approach that utilizes a framework or theory to guide the analysis. A 5-step approach was developed based on previous studies [27–29], including (i) familiarization, (ii) indexing and coding, (iii) developing new codes, (iv) charting, and (v) identifying themes. At first, each coder conducted multiple reviews of the transcripts to familiarize themselves with the data and gain a deep understanding of the data. In step 2, the coders highlighted those parts of the text that, on first impression, appeared to be related to the predetermined codes based on the CFIR framework. The highlighted texts were then coded accordingly. Two coders coded 10 transcripts first to achieve consensus on coding. They discussed the coding process. When disagreement occurred, the original transcript was referred to understanding participants' meanings. After achieving consensus on coding for the first 10 transcripts, they coded all transcripts independently. After all transcripts were coded, the two coders reviewed the coding results, and any discrepancies were resolved through discussions. In step 3, any text that could not be categorized with the initial coding scheme would be given a new code. The data collection process continued until data saturation–when adding further data showed no new information and the extra collected data were redundant. When the new code was not produced in the last three interviews, saturation was achieved and data collection stopped. This process of achieving saturation was used in previous studies [30, 31]. In step 4, once all the data had been coded, the text from transcripts for each participant and codes were abstracted and inserted into the corresponding cell in the data matrix. Finally, similar verbatim words/sentences (meaning units) representing the same idea were grouped to form a theme.

### Quantitative study

A cross-sectional survey study was conducted among physicians and nurses in primary care settings from July to October 2022.

#### Participants and data collection

**Fig. 1:**
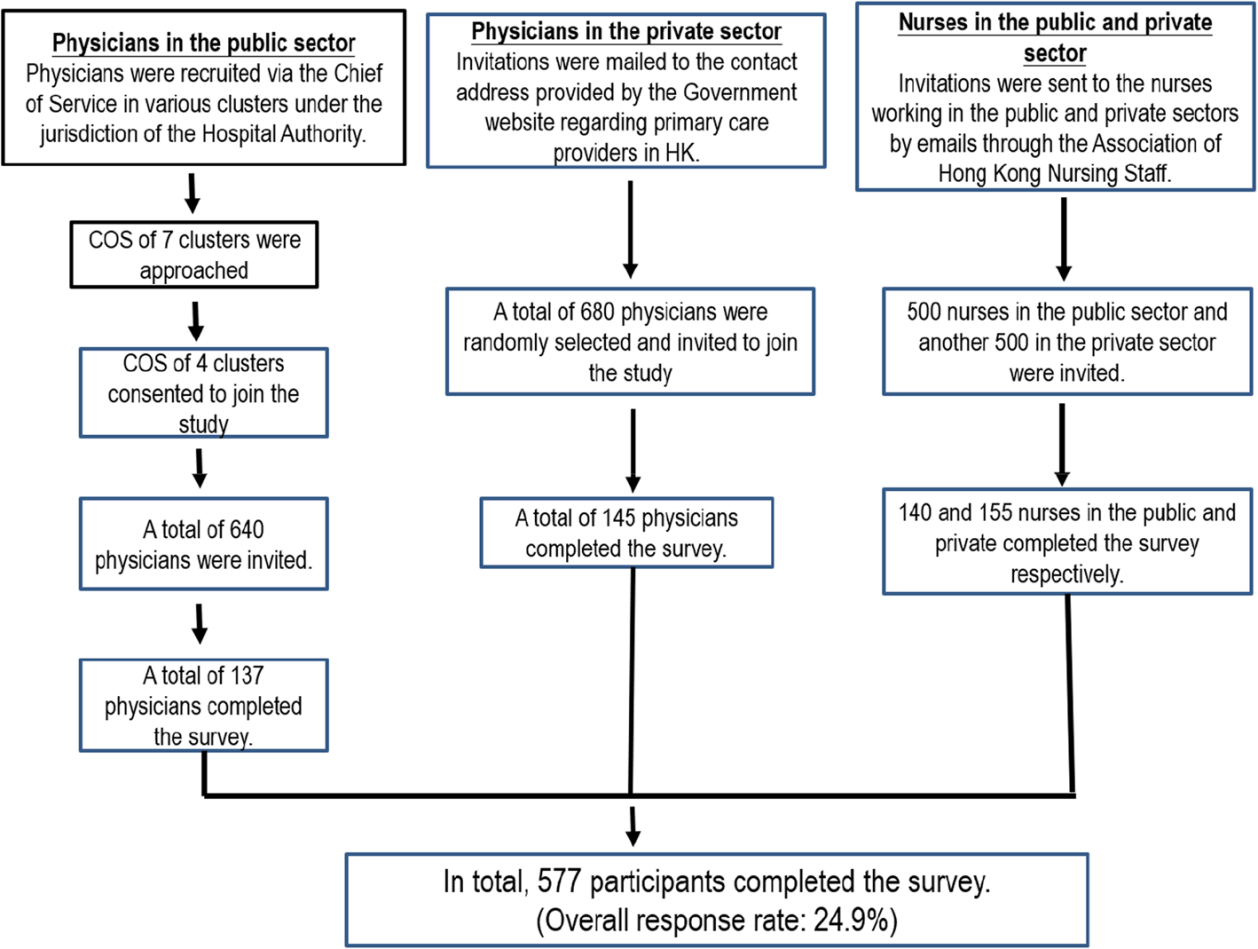
Flowchart of participant recruitment

The inclusion and exclusion criteria were the same as those in the qualitative study. A flowchart of participant recruitment was shown in Fig. 1. Upon completion of the survey. Each participant received a supermarket coupon of HK$50 (US$6.5) as a token of appreciation.

##### Physicians in the public sector

All public hospitals in Hong Kong are managed by the Hospital Authority. They are organized into seven hospital clusters based on their locations [32]. We approached all chiefs of service of these seven clusters and four of them were willing to support our study. Facilitated by the chiefs of service, a link to access an online questionnaire was sent to physicians working in the General Out-patient Clinics, and Family Medicine Integrated/Specialist Clinics within the clusters through email. Before starting the online survey, participants read a statement indicating that participation was voluntary, refusal to participate would have no effect on them, and the survey would not collect personal identifying information. Online informed consent was obtained. Among 640 physicians in the public sector being invited, 137 completed the survey (response rate: 21.4%).

##### Physicians in the private sector

A list of all physicians (4511 in total) working in the primary care settings in the private sector was retrieved from the government webpage [33] and entered into an Excel file. Using the function of selecting random cells, 680 physicians were randomly selected from the list. Hard copies of the questionnaires with a quick response (QR) code to access the same online questionnaire were sent to their contact addresses. Participants could return hard copies of the questionnaire with the written consent form by mail, or provide online consent and complete the online questionnaire. A total of 145 physicians in the private sector completed the survey, the response rate was 21.3%.

##### Nurses

The Association of Hong Kong Nursing Staff keeps the contact information of all nurses who applied for membership in the Association in Hong Kong. Facilitated by the Association, 500 nurses working in the public sector and another 500 nurses working in the private sector in the primary care settings were randomly selected from their database. A link to access the same online questionnaire was sent to them through email. With online informed consent, 140 (response rate: 28%) and 155 (response rate: 31%) nurses in the public and private sectors completed the online survey, respectively.

#### Measures

##### Development of the questionnaire

The same panel developed the questionnaire. The qualitative study identified 22 constructs of CFIR that were relevant to SBI implementation among primary care providers in Hong Kong. The panel adapted validated measurements and constructed questions to measure these constructs. The questionnaire was pilot tested among five physicians and five nurses to assess its clarity and readability. All the physicians and nurses in the pilot study indicated that the items of the questionnaire were easy to understand and the length of the questionnaire was acceptable. These physicians and nurses did not participate in the actual survey. The panel finalized the questionnaire based on their comments.

We created the online questionnaire using Qualtrics, a commonly used online survey platform. The survey consisted of 71 items (approximately 10 items per page for 7 pages) and required around 20 minutes to complete. The Qualtrics performed a completeness check before the questionnaire was submitted. Participants were able to review and change their responses using a Back button. All data were stored in the Qualtrics server and protected by a password.

##### Background characteristics

Participants reported background information which included gender, age, number of years working in the current workplace, current employment status, training status in the Hong Kong Academy of Medicine (for physicians), job position/rank (for nurses), public/private sector, and whether they drank alcohol in the past year.

##### SBI implementation

Participants were asked to estimate the proportion of patients they screened for alcohol consumption using the AUDIT and gave standard intervention according to the SBI protocol in the past year (response categories: 0%, 1-25%, 26-50%, 51-75%, >75%). SBI implementation was defined as providing both screening using AUDIT and standard interventions based on SBI protocol.

##### CFIR constructs

We measured 22 constructs under all five CFIR domains. Details of the measurements were presented in Supplementary Tables 1 and 2. Reliabilities measured by Cronbach’s alpha of the scales measuring CFIR constructs were acceptable (0.65-0.85) (Supplementary table 1).

##### Attitudes toward drinking in the Chinese culture

We added attitudes toward drinking in the Chinese culture since the participants in the qualitative interview study reported that some beliefs in the Chinese culture might affect people’s drinking. We adapted item/subscale from the validated Chinese version of the Cultural Attitudes Toward Drinking Scale (CADS) to measure social approval for drinking (1 item) and functions of drinking (5 items) [34]. The Cronbach’s alpha of the Functions of Drinking subscale was 0.79. In addition, two single items were constructed to measure the influence of moderation on drinking proposed by Confucian and Taoist philosophies and the stigma-related issue of addressing drinking problems (response categories were 1=strongly disagree, 2=disagree, 3=neutral, 4=agree, 5=strongly agree).

#### Sample size planning

There were four groups of participants (physicians or nurses in the public or private sectors). The target sample size is 150 per group. As an illustration of the statistical power, given a power of 0.8 and an alpha of 0.05, such sample size could detect the smallest between-group difference of 11.8% for SBI implementation, assuming 10-30% implemented SBI in the reference group. Assuming 10-30% in the reference group (without a facilitator) implemented SBI, the sample size (n=150) would give the smallest ratio of 2.53 between those with and without such a facilitator (PASS 11.0, NCSS, LLC).

#### Statistical analysis

Given the differences in roles of sectors and professions, the analyses were conducted separately for the public versus private sector and physicians versus nurses. Differences in SBI implementation and CFIR constructs between physicians/nurses in the public sector and the private sector were compared using logistic regression or ANCOVA, after adjusting background characteristics with significant between-group differences. Using SBI implementation in the last year as the dependent variable, and background characteristics as independent variables, odds ratio (OR) predicting the dependent variable were obtained using logistic regression models. After adjusting those sociodemographic characteristics with *p*<0.05 in the univariate analysis, the associations between the independent variables of interest (e.g., CFIR constructs) and the dependent variable were then obtained by adjusted odds ratio (AOR), and respective 95% confidence interval (CI) were derived from the analyses. Each AOR was obtained by fitting a single logistic regression model, which involved one of the independent variables and the significant sociodemographic variables. SPSS version 26.0 (Chicago, IL, United States) was used for data analysis, and *p*<0.05 were considered as statistically significant.

## Results

### Qualitative study

#### Background characteristics

A total of 25 physicians and 24 nurses were approached, of which five physicians and three nurses refused to participate due to unavailability or lack of interest in the study. Finally, 20 physicians and 21 nurses completed the interviews. Nearly half of them were female (51.2%) and worked in the public sector (51.2%). The majority were aged 20-40 years (78.1%) (Table 1).

**Table 1:** Background characteristics of the participants in the qualitative study (*N*=41)

	Physicians	Nurses	Total
	N (%)	N (%)	N (%)
Gender			
Male	10 (50.0)	10 (47.6)	20 (48.8)
Female	10 (50.0)	11 (52.4)	21 (51.2)
Age			
20-30	9 (45.0)	8 (38.1)	17 (41.5)
30-40	7 (35.0)	8 (38.1)	15 (36.6)
40-50	4 (20.0)	5 (23.8)	9 (22.0)
Sector			
Public	10 (50.0)	11 (52.4)	21 (51.2)
Private	10 (50.0)	10 (47.6)	20 (48.8)
Training status in the Hong Kong Academy of Medicine (Physicians)			
Nil	4 (20.0)	NA	NA
Basic trainee	3 (15.0)	NA	NA
Higher trainee	5 (25.0)	NA	NA
Academy fellow	8 (40.0)	NA	NA
Job position (Nurses)			
Enrolled nurse	NA	8 (38.1)	NA
Registered nurse	NA	9 (42.9)	NA
Advanced practice nurse	NA	4 (19.0)	NA

*NA* Not applicable

#### CFIR determinants of the implementation of SBI

Sample quotes of the interviews were presented in Table 2.

**Table 2:** Sample quotes of the interviews in the qualitative study

Domains	Constructs	Sample quotes
Intervention characteristics	Evidence strength and quality	*“I don’t know much about the efficacy of SBI. Is the evidence about one-off SBI given to patients or several sessions are needed? If several sessions are needed, how long should be the interval between every two sessions? I don’t think the patient would come to the clinic just to receive SBI. More importantly, we do not ask patients to return for a follow-up visit only to check how they have managed to cut down on their alcohol intake.” (Nurse 3, public sector)* *“I read some articles about SBI before. It was supported by some randomized controlled trials. Its effects on reducing alcohol consumption among heavy drinkers are good. Therefore, I used it when appropriate.” (Physician 11, private sector)*
	Relative advantage	*“There are too many questions and steps in SBI which makes it less advantageous or flexible than my own approach. I tend to use CAGE questions and also prefer to have conversations with the patients rather than a questionnaire.” (Nurse 17, private sector)*
	Adaptability	*“SBI does not fit well with my routine practice. For my 10-year experience, I get the information from my patients without using a questionnaire. As it is called screening, I am not comfortable to ask every patient for at least three items in the AUDIT, let alone the 10 items in total. Additionally, the existing computer system does not have a recording system for SBI. My work is not organized for doing such assessment or intervention.” (Physician 6, public sector)* *“I do not agree that every patient should be screened. On one hand, I think I am the right person to ask. On the other hand, I am also trying to figure out whether it is appropriate to ask when a patient comes to me. If every patient is given SBI, it is like a health promotion, not a consultation.” (Physician 14, public sector)*
	Complexity	*“Overall, the procedures of doing SBI are easy to understand and it looks simple. However, when looking at it in detail, it is not that easy. We need to calculate the alcohol unit intake per day for the patients. This may be a bit complicated since different kinds of alcohol contain different alcohol contents and some patients may not be able to report accurately how much they have drunk.” (Physician 17, private sector)*
	Cost	*“I am already stressed in my daily work. It would be great if I only need to deal with alcohol issues for a single consultation. Nonetheless, I have patients with multiple problems. Adding extra work to my existing duty will make me extremely busy since my work schedule is already fully occupied.” (Physician 16, public sector)* *“If I am required to use this tool, it seems that I will just try to get this job done. Something just like paperwork that needs to be documented. I would say, this increases extra time on administration and management.” (Nurse 8, public sector)*
Outer setting	Patient needs	*“When I ask my patients about their drinking, they are willing to provide any information I want to know. They understand that this is part of the consultation. They have the expectation that providing more information could let us come up with better treatments. Therefore, I don’t feel embarrassed or difficult to ask.” (Physician 4, private sector)* *“If the patient comes to the clinic and his/her symptoms, for example, sleeping problems, diabetes, and hypertension, make me feel that alcohol is part of the health problem. It is easy for you to ask about their lifestyle issues, such as ‘Do you smoke? Do you drink?’ I avoid going straight for it by asking ‘Do you drink?’ but combine it with questions related to other lifestyles so it seems less awkward. I don’t hesitate to ask the patient such questions because I want to help them by letting them know how their lifestyle would affect their health status.” (Physician 17, private sector)*
	Cosmopolitanism	*“There are various treatment service centres, such as Hospital Authority’s Psychiatry Specialist Out-patient Clinics, Tung Wah Group of Hospitals Alcohol Abuse Prevention and Treatment Service, etc. The thing I concern is about the capacity of their treatment services. Since the waiting list of existing demand is long, let alone new cases discovered by SBI. ” (Nurse 1, private sector)*
	External policy and incentives	*“I don’t think the government offers good support for us to provide prevention services. No good opportunities or resources are available. Practicing prevention takes time. I would suggest more doctors and nurses should be trained and employed, and as well as more clinics or hospitals should be built in order to alleviate our workload and as a result being able to spend more time on prevention.” (Nurse 12, public sector)* *“As I know, a few years ago, only a few training sessions were provided by the Department of Health. This is not enough. More promotion of SBI and training sessions should be provided.” (Nurse 9)* *“Compared to drinking, it is easier for you to find smoking campaigns in the media and they really bring up the public health issue of smoking. Unfortunately, we do not see this with regard to alcohol. On the other hand, the message of drinking is often linked to safe driving issues, but not related to health issues in promotion campaigns in Hong Kong.” (Physician 5, public sector)*
Inner setting	Networks and communication	*“For smoking, there is at least one specialist staff responsible for the smoking issue in many clinics in the public sector. However, there is no such service for alcohol drinking. Interprofessional cooperation is crucial. For instance, interventions would take much longer time than screening. If we do the screening and refer the patients to another specialist in our clinic, this would save us a lot of time.” (Physician 12, public sector)*
	Tension for change	*“I don’t think there is a strong need to implement SBI right now to help my patients. For my 20-year experiences as a doctor, alcohol issue is not a critical issue for most of the patients.”(Physician 9, private sector)* *“Since it is not required in my work and it is not one of the quality indicators, I prefer not to do it since it is time-consuming.” (Physician 10, private sector)*
	Relative priority	*“As you know, many patients usually have multiple presenting problems. I have to prioritise, and there are more important issues to be addressed, such as smoking, blood pressure, or other chronic diseases.” (Nurse 12, public sector)*
	Leadership engagement	*“Our supervisors seldom talked about alcohol-related issues, let alone SBI. More concrete instructions or support should be provided by our supervisors.” (Nurse 20, private sector)*
	Organizational culture	*“We have a protocol to carry out our job duties. It is not easy, or even impossible to adopt a new approach to treating patients in my clinic, unless it is approved by your supervisors, department heads, and even the Quality and Safety Unit. This is the case for both innovative approaches or evidence-based methods.” (Nurse 10, public sector)*
	Goals and feedback	*“As mentioned, there is a lack of concrete plans, resulting in a lack of goals for implementing SBI in my clinic. Worse still, there is no feedback, both formal and informal, from our supervisors or senior colleagues, or the performance reviews.” (Nurse 6, private sector)*
	Available resources	*“I don’t notice if there is any training provided. It is only a guideline available online. Honestly, we would not take the initiative to go into the details of the guideline. It would be better if there is a video demonstrating how to use SBI. Unfortunately, I could not find any training video, not to mention the physical training. Things will not happen by themselves.” (Nurse 11, public sector)* *“Compared to smoking, there are relatively fewer resources about health information related to alcohol drinking for patients, such as pamphlets. If there are plenty of such resources for the patients in the clinic, it is easier for us to touch upon their alcohol consumption.” (Nurse 6, private sector)*
Characteristics of individuals	Knowledge about the intervention	*“I usually use the CAGE questions, I don’t know much about SBI recommended by the Department of Health.”(Physician 2, private sector)* *“When studying in medical school, alcohol was presented through certain types of health conditions, such as liver cirrhosis, liver cancers and gastric ulcers. I was taught how to use medications to treat patients with alcohol problems. However, there was no introduction to something like screening questionnaires or brief interventions.” (Physician 19, public sector)*
	Belief about the intervention	*“If the patients drink very often, we have the responsibility to inform them about the harms of heavy drinking. Hopefully, patients also understand that we care and we want to help them.” (Nurse 9, private sector)* *‘I don’t think using SBI is a rewarding task as it takes time. Furthermore, the patients’ drinking behavior may not change in a short time. It needs much effort for this to happen.” (Physician 18, public sector)*
	Self-efficacy	*“When it comes to behavior change, it is very challenging. Although there are many useful theories or practical skills to help patients change their unhealthy behaviors including drinking, I do not think that I am powerful enough to influence them. Although the procedures are easy to understand, I am not confident in using it to help patients in my daily work since it takes time. Therefore, I don’t think I can make a difference.” (Nurse 15, public sector)*
Process	Planning	*“Several years ago, I heard that there was a discussion about the possibility to implement SBI in our daily work. However, due to some reasons, I am not sure… there are no concrete plans.” (Nurse 3)* *“No clear guidelines about doing SBI in my clinic. If it is not required in my work, I will not do it since I have already too many tasks waiting for me.” (Nurse 18, private sector)*
	Engaging	*“I did not see there are any advocates in my clinic who actively support or promote SBI. This may lead to a low interest or motivation in using SBI.” (Nurse 17, private sector)*
	Executing	*“Although our mission is to help manage patients’ health conditions and alcohol consumption leads to lots of diseases, low use of SBI, honestly, does not align with our mission or strategic plan.” (Physician 20, private sector)*
	Reflecting and evaluating	*“There is really no meeting about how we have performed SBI in the clinic. For instance, I don’t know how our staff have used it in their daily work.” (Nurse 2, public sector)*
Additional new code	Social context (1): The value of moderation proposed by Confucianism and Taoism in controlling drinking in the Chinese context	*“People should not drink in excess amount in any situation. Just like the concept of moderation in the Chinese culture that every behavior should not be done in excess.” (Nurse 9, private sector)*
	Social context (2): The stigmatization of alcohol dependence	*“Some patients may refuse to admit they have alcohol problems. They don’t want to be labeled as ‘alcoholic’ or ‘alcohol ghost’. There is some kind of shame linked with it. This word represents not only addiction, but also madness, disorganized rudeness, and many other kinds of negative attributes. Therefore, some patients may not want to admit having a drinking problem, especially older people. Because this term is more popular in the old days.” (Physician 4, private sector)* *“Treatment services are provided by psychiatric services or drug addiction centres. Many patients worry that they may be perceived as having mental health problems or people taking illegal drugs. Although they are willing to answer your questions regarding alcohol drinking, they are reluctant to accept it as a serious problem. Patients who usually drink in social events regard it as part of their life.” (Physician 12, public sector)*

**Intervention characteristics**

*Evidence strength and quality*

Some participants were unsure about the efficacy of SBI (15/41 participants). They expressed their concerns about how much the patients would benefit from receiving SBI (10/41 participants). They would need concrete evidence before making decisions on how much effort should be put into this work (9/41 participants). On the other hand, some participants said that they felt confident to use SBI as they knew that the efficacy of SBI was supported by randomized controlled trials (19/41 participants).

*Relative advantage*

Participants reported that they mainly relied on their previous experiences and knowledge in order to address alcohol use with the patients (30/41 participants). They did not think that SBI was more effective than their own approach (20/41 participants). Several of them used the CAGE (Cut, Annoyed, Guilty, Eye-opener) questions [35] (8/41 participants). A few participants admitted that using a standard tool, like the AUDIT, was good for record-keeping and follow-up in the future (7/41 participants).

*Adaptability*

Participants thought that SBI did not fit well with the way they work in the clinic and felt it was inappropriate to screen all patients in general practice (25/41 participants). There would be interruptions in their routine work (24/41 participants).

*Complexity*

Although the procedures of doing SBI were easy to understand, the participants thought that it was complex to some extent, especially calculating alcohol units intake (20/41 participants). Some participants reported that assessing patients’ stage of change and giving different types of brief interventions were also complicated (25/41 participants).

*Cost*

There was consensus that using SBI was time-consuming, especially if the patients were heavy drinkers and standardized advice or interventions were needed (32/41 participants). They should also document the patients’ answers and what sort of advice they had been provided (15/41 participants). This would further increase their burden in terms of administrative or management costs (24/41 participants).

**Outer setting**

*Patient needs*

The participants agreed that their patients were willing to answer questions related to alcohol consumption and they believed that the patients thought that the physicians/nurses cared about them by asking such questions (25/41 participants). If the patients showed certain specific symptoms or signs related to alcohol drinking, they acted as a trigger for discussion of lifestyle issues including alcohol drinking (23/41 participants).

*Cosmopolitanism*

Participants reported that it was easy to refer patients to specialized addiction care/ treatment or community alcohol service (27/41 participants). They knew some referral services were available both provided by the government or community bodies (32/41 participants). On the other hand, they suggested that such referral services should be increased to reduce the waiting time for patients in order to receive timely treatment (26/41 participants).

*External policy and incentives*

The participants reported that there was no government policy to support preventive medicine in primary care settings, such as using SBI (26/41 participants). Additionally, the lack of training on SBI provided by the government, and the lack of public education campaigns were also reported as barriers (19/41 participants).

**Inner setting**

*Networks and communication*

Participants reported that the staff in the clinic did not have expectations of each other to use SBI (25/41 participants). Additionally, a lack of inter-professional cooperation in the delivery of SBI in their clinic was reported as a barrier (29/41 participants). The participants expressed a need for health professionals or social workers who were responsible for alcohol issues in their workplace (28/41 participants).

*Tension for change*

While some participants expressed there was a need for using SBI, especially those in the public sector (15/41 participants), other participants deemed that there was no tension about implementing SBI right now (24/41 participants). They thought that alcohol was not a critical issue for most of their patients (27/41 participants). Additionally, SBI was only recommended by the Department of Health but was not a must (17/41 participants).

*Relative priority*

Low priority for alcohol issues was reported (29/41 participants). Participants noted that patients usually have several health problems and there are always competing demands, leaving limited time for alcohol issues (26/41 participants).

*Leadership engagement*

Participants reported that their supervisors/managers/senior colleagues seldom talked about SBI with them (25/41 participants). There was a lack of concrete instructions, encouragement, or support to help patients reduce drinking (31/41 participants).

*Organizational culture*

The staff was not willing to make changes in their routine work using innovative approaches, even evidence-based practices (18/41 participants). If they were not required or did not have clear instructions to do it, they would not do it in the end (25/41 participants). On the other hand, participants believed that most of the staff had a sense of cooperation to provide high-quality care to the patients (27/41 participants).

*Goals and feedback*

The participants did not notice any goals of using SBI in their workplace (28/41 participants). They did not receive feedback from their supervisors or work reports on how they performed (30/41 participants).

*Available resources*

Participants reported that there was a lack of printed SBI materials or health information related to alcohol drinking for patients in the workplace (22/41 participants). They also expressed that there was no training provided in their workplace (29/41 participants). Participants wondered that although SBI was highly recommended by the government, there was a lack of training for implementing SBI in the clinic (25/41 participants).

**Characteristics of individuals**

*Knowledge about the intervention*

Although some participants said that they knew very well about SBI (23/41 participants), a lack of knowledge or low awareness of SBI recommended was still reported as a barrier by some participants (13/41 participants). Additionally, participants expressed a lack of alcohol training when they studied in medical school (8/41 participants).

*Belief about the intervention*

The belief that they have the responsibility and right to ask their patients about drinking was reported as a facilitator (17/41 participants). They viewed this task as within their role working in the primary care settings (18/41 participants). However, they did not think using SBI was rewarding because it takes time and the change in the patient’s alcohol consumption may also take a long time to occur (12/41 participants).

*Self-efficacy*

Having confidence in using SBI was reported by participants who stated using SBI in the past few months (13/41 participants). Some participants expressed a lack of confidence in using the SBI to change the patient’s drinking behavior (14/41 participants). Such issues include using the AUDIT to screen patients and explaining risks to health from different levels of alcohol consumption. They could offer many suggestions to the patients but their efforts did not necessarily lead to anything good to happen (7/41 participants).

**Process**

*Planning*

Most participants reported there were no concrete plans for implementing SBI. The lack of a plan implied a lack of guidelines for implementing the SBI (34/41 participants).

*Engaging*

Participants reported a lack of champions for SBI, who actively support and promote SBI. The staff generally did not take an active interest in implementing SBI (35/41 participants).

*Executing*

Participants also reported a lack of consistency in implementing SBI that was aligned with the clinic’s mission and strategic plan (26/41 participants).

*Reflecting and evaluating*

There was a lack of good communication about how different changes are related to SBI, e.g., frequency of using SBI among staff, and a lack of data to guide their clinic to implement SBI (e.g., performance reviews, and assessments) (33/41 participants).

#### Additional new code

##### Social context

Based on our data analysis, two themes could not be classified into any of the CFIR constructs. A new code, social context, was created.

*The value of moderation proposed by Confucianism/ Taoism to control drinking*

Some participants reported that it was important that people should control the amount of alcohol they drank in any situation (17/41 participants). They believed that every behavior should not be done to an excess amount, which is consistent with the ideas of moderation, proposed by Confucianism/Taoism, a philosophy and ethical system that has had a significant influence on Chinese culture.

*Stigmatization of alcohol dependence*

Some participants thought that it was difficult for the patients to admit they had alcohol problems or dependence, making it challenging to discuss alcohol issues with them (15/41 participants). In Hong Kong, there was the term ‘alcohol ghost’, in Cantonese ‘酒鬼zau2 gwai2’. Simply translated to English, this word describes a person with alcohol problems as just like a ghost. Patients had the fear of being labeled in such a way. Additionally, alcohol problems were treated in psychiatric services or drug addiction treatment centres. Patients avoided the perceived stigma of mental health problems or mixing with drug addicts (15/41 participants).

### Quantitative study

#### Background characteristics

Background characteristics of physicians and nurses were presented in Table 3. As compared to physicians in the public sector, those in the private sector were older (>50 years: 34.5% versus 10.2%, *p*<0.001), working in the current clinic for a longer time (>20 years: 23.4% versus 5.1%, *p*<0.001), more likely to be part-time employed (14.5% versus 6.6%, *p*=0.03) and without training in HKAM (51.0% versus 0%, *p*<0.001). As compared to nurses in the public sector, those in the private sector had longer service time in the current clinic (>20 years: 8.4% versus 2.9%, *p*=0.004) (Table 3).

**Table 3:** Background characteristics of the participants in the quantitative study (*N*=577)

	Physicians			Nurses		
	Public (*N*=137)	Private (*N*=145)	*P* values	Public (*N*=140)	Private (*N*=155)	*P* values
	n (%)	n (%)		n (%)	n (%)	
Gender						
Male	82 (59.9)	85 (58.6)		18 (12.9)	21 (13.5)	
Female	55 (40.1)	60 (41.4)	0.83	122 (87.1)	134 (86.5)	0.86
Age (years)						
≤30	23 (16.8)	6 (4.1)		39 (27.9)	47 (30.3)	
31-40	67 (48.9)	36 (24.8)		69 (49.3)	59 (38.1)	
41-50	33 (24.1)	53 (36.6)		22 (15.7)	30 (19.4)	
>50	14 (10.2)	50 (34.5)	**<0.001**	10 (7.1)	19 (12.3)	0.19
Years of working in the current workplace (years)						
≤5	42 (30.7)	42 (29.0)		42 (30.0)	52 (33.5)	
6-10	45 (32.8)	24 (16.8)		69 (49.3)	46 (29.7)	
11-15	33 (24.1)	53 (36.6)		19 (13.6)	29 (18.7)	
16-20	18 (13.1)	18 (12.4)		6 (4.3)	15 (9.7)	
>20	7 (5.1)	34 (23.4)	**<0.001**	4 (2.9)	13 (8.4)	**0.004**
Current employment status						
Full-time	128 (93.4)	124 (85.5)		133 (95.0)	140 (90.3)	
Part-time	9 (6.6)	21 (14.5)	**0.03**	7 (5.0)	15 (9.7)	0.13
Training status in HKAM (Physician)						
Nil	0	74 (51.0)		NA	NA	
Basic Trainee	16 (11.7)	0		NA	NA	
Higher Trainee	22 (16.1)	0		NA	NA	
Academy Fellow	99 (72.3)	71 (49.0)	**<0.001**	NA	NA	NA
Job position (Nurse)						
Enrolled nurse	NA	NA		76 (54.3)	87 (56.1)	
Registered nurse	NA	NA		46 (32.9)	54 (34.8)	
Advanced practicenurse	NA	NA	NA	18 (12.9)	14 (9.0)	0.57
Type of practice/work setting						
Clinic where physician practice as solo	NA	54 (37.2)		NA	33 (21.3)	
Clinic where with partners/ group practice	NA	57 (39.3)		NA	61 (39.4)	
General Outpatient Clinic	99 (72.3)	18 (12.4)		107 (76.4)	35 (22.6)	
Family Medicine Integrated/Specialist Clinic	38 (27.7)	16 (11.0)	**<0.001**	33 (23.6)	26 (16.8)	**<0.001**
Drinking in the past year						
Yes	78 (56.9)	82 (56.6)		51 (36.4)	68 (43.9)	
No	59 (43.1)	63 (43.4)	0.95	89 (63.6)	87 (56.1)	0.19

*P* values were obtained by chi-squared test

*NA* Not applicable

#### SBI implementation

After controlling for background characteristics with significant between-group differences, there was no significant difference in SBI implementation in the past year between physicians/nurses in public and private sectors (physicians: 16.8% versus 14.5%, p=0.93; nurses: 30% versus 26.5%, p=0.53) (Table 4). Among physicians and nurses who had performed SBI in the past year, they only provided it to 1-25% of their patients.

**Table 4:** Descriptive statistics of the measurement (N=577)

	Physicians			Nurses		
	Public (*N*=137)	Private (*N*=145)		Public (*N*=140)	Private (*N*=155)	
	N (%)	N (%)	Adjusted *P* value*	N (%)	N (%)	Adjusted *P* value
**Performed SBI in the past year**						
Yes	23 (16.8)	21 (14.5)		42 (30.0)	41 (26.5)	
No	114 (83.2)	124 (85.5)	.93	98 (70.0)	114 (73.5)	.53
	Mean^+^ (SD^+^)	Mean^+^ (SD^+^)	Adjusted *P* values^#^	Mean^+^ (SD^+^)	Mean^+^ (SD^+^)	Adjusted *P* values^#^
**Intervention characteristics**						
Evidence Strength Scale ^a^	3.67 (0.52)	3.58 (0.60)	0.81	3.32 (0.56)	3.36 (0.64)	0.63
Relative advantage	3.48 (0.59)	3.41 (0.70)	0.66	3.34 (0.68)	3.36 (0.72)	0.68
Adaptability Scale ^b^	3.07 (0.63)	3.51 (0.57)	**0.02**	3.33 (0.69)	3.66 (0.65)	**0.03**
Complexity Scale ^c^	3.32 (0.65)	3.27 (0.62)	0.72	3.31 (0.57)	3.33 (0.60)	0.73
Cost	3.92 (0.87)	3.36 (0.83)	**0.008**	3.86 (0.73)	3.32 (0.79)	**0.01**
**Outer setting**						
Cosmopolitanism	3.17 (0.88)	3.16 (0.87)	0.60	2.93 (0.82)	3.21 (0.89)	**0.03**
Patient Needs Scale ^d^	3.26 (0.44)	3.25 (0.45)	0.50	3.11 (0.39)	3.12 (0.50)	0.66
External policy or incentives	3.58 (0.93)	3.19 (0.89)	0.05	3.18 (0.79)	3.26 (0.81)	0.88
**Inner setting**						
Relative priority	2.67 (0.93)	2.61 (0.80)	0.69	2.60 (0.79)	2.62 (0.88)	0.80
Tension for change	2.81 (0.83)	2.39 (0.87)	**0.03**	2.95 (0.90)	2.87 (0.89)	0.66
Network and communication	3.29 (0.71)	3.13 (0.81)	0.38	3.31 (0.74)	3.31 (0.81)	0.97
Leadership engagement	3.36 (0.73)	3.04 (0.88)	**0.04**	3.44 (0.75)	3.34 (0.87)	0.51
Organizational Culture Scale ^e^	3.70 (0.57)	3.51 (0.65)	0.49	3.58 (0.53)	3.46 (0.61)	0.15
Goals and Feedback Scale ^f^	2.51 (0.73)	2.41 (0.66)	0.29	2.49 (0.74)	2.54 (0.71)	0.09
Available Resources Scale ^g^	2.69 (0.70)	2.30 (0.68)	**0.02**	2.75 (0.63)	2.31 (0.67)	**0.01**
**Characteristics of individuals**						
Knowledge Scale ^h^	3.33 (0.58)	3.23 (0.73)	0.78	3.20 (0.61)	3.18 (0.72)	0.96
Positive Beliefs Scale ^i^	3.37 (0.38)	3.30 (0.53)	0.50	3.15 (0.45)	3.24 (0.47)	0.18
Negative Beliefs Scale ^j^	2.89 (0.56)	2.82 (0.63)	0.65	3.08 (0.49)	2.98 (0.54)	0.20
Self-efficacy Scale ^k^	2.93 (0.52)	2.89 (0.62)	0.39	2.90 (0.58)	3.06 (0.64)	0.10
**Process**						
Planning Scale ^l^	2.49 (0.72)	2.61 (0.74)	0.57	2.97 (0.77)	2.94 (0.76)	0.78
Engaging Scale ^m^	2.81 (0.73)	2.84 (0.74)	0.34	2.87 (0.70)	2.74 (0.77)	0.30
Executing	2.59 (0.88)	2.84 (0.92)	0.26	2.72 (0.97)	2.70 (0.95)	0.14
Reflecting and Evaluating Scale ^n^	2.75 (0.72)	2.76 (0.77)	0.41	2.84 (0.74)	2.76 (0.73)	0.15
**Cultural attitudes toward drinking**						
Social approval of drinking	3.91 (0.96)	3.80 (0.99)	0.11	3.69 (0.82)	4.01 (0.88)	0.15
Functions of Drinking in the Chinese Culture Scale ^o^	3.67 (0.53)	3.65 (0.56)	0.10	3.44 (0.55)	3.55 (0.54	0.13
Value of moderation proposed by Confucianism/ Taoism to control drinking	4.17 (0.76)	4.18 (0.83)	0.53	3.79 (0.73)	3.70 (0.76)	0.75
Stigmatization issue of alcohol use	2.58 (0.77)	3.01 (0.89)	0.05	3.19 (0.77)	3.31 (0.73)	0.44

*SD* Standard deviation

+Scale scores were standardized by dividing total scores by number of items

^*^Adjusted *P* value was obtained by logistic regression after adjustment for background characteristics with significant between-group differences listed in Table 3

^#^Adjusted *P* values were obtained by ANCOVA after adjustment for background characteristics with significant between-group differences listed in Table 3

^a^Evidence strength Scale: three items, Cronbach’s alpha: 0.85

^b^Adaptatibility Scale: two items, Cronbach’s alpha: 0.79

^c^Complexity Scale: four items, Cronbach’s alpha: 0.78

^d^Patient Needs Scale: five items, Cronbach’s alpha: 0.76

^e^Organizational Culture Scale: four items, Cronbach’s alpha: 0.85

^f^Goals and Feedback Scale: two items, Cronbach’s alpha: 0.76

^g^Available Resources Scale: four items, Cronbach’s alpha: 0.85

^h^Knowledge Scale: two items, Cronbach’s alpha: 0.82

^i^Positive Beliefs Scale : four items, Cronbach’s alpha: 0.65

^j^Negative Beliefs Scale: three items, Cronbach’s alpha: 0.74

^k^Self-efficacy Scale: six items, Cronbach’s alpha: 0.68

^l^Planning Scale: two items, Cronbach’s alpha: 0.83

^m^Engaging Scale: two items, Cronbach’s alpha: 0.78

^n^Reflecting and Evaluating Scale: two items, Cronbach’s alpha: 0.81

^o^Functions of drinking in the Chinese Culture Scale: five items, Cronbach’s alpha: 0.84

#### CFIR constructs

The mean and standard deviation (SD) of scales/items of CFIR constructs were presented in Table 4. A higher score indicated higher perception of this factor being present/relevant (Supplementary table 3). As compared to physicians/nurses in the private sector, those working in the public sector believed that SBI fit less well in their clinics (physicians: *p*=.02, nurses: *p*=0.03), perceived a higher cost to implement SBI (physicians: *p*=.008, nurses: *p*=.01), but had more available resources to implement SBI (physicians: *p*=0.02, nurses: *p*=0.02). Physicians in the public sector perceived a higher need to implement SBI (tension for change) (*p*=0.03) and better leadership engagement (*p*=0.04), as compared to those in the private sector. Nurses in the private sector perceived that it was easier to refer patients with alcohol problems (*p*=.03) compared to nurses in the public sector (Table 4).

#### Factors associated with SBI implementation

Physicians and nurses who were older were less likely to implement SBI compared to their younger counterparts. Nurses working in the public sector for a shorter time were more likely to implement SBI. In the private sector, registered nurses were more likely to implement SBI than enrolled nurses (Table 5).

**Table 5:** Associations of sociodemographic characteristics and the use of SBI (both AUDIT and BI) at least one episode in the past year (*N*=577)

	Physicians				Nurses			
	Public (*N*=137)		Private (*N*=145)		Public (*N*=140)		Private (*N*=155)	
	OR (95%CI)	*P* values	OR (95%CI)	*P* values	OR (95%CI)	*P* values	OR (95%CI)	*P* values
Gender								
Male	1.00		1.00		1.00		1.00	
Female	0.76 (0.30,1.94)	0.57	0.85 (0.33,2.20)	0.74	0.48 (0.18,1.33)	0.16	0.68 (0.25,1.83)	0.44
Age (years)								
≤30	1.00		1.00		1.00		1.00	
31-40	0.26 (0.09,0.73)	**0.01**	0.16 (0.03,1.03)	0.05	0.31 (0.14,0.71)	**0.006**	0.32 (0.13,0.74)	**0.008**
41-50	0.04 (0.01,0.35)	**0.004**	0.21 (0.04,1.18)	0.08	0.21 (0.06,0.74)	**0.02**	0.25 (0.08,0.76)	**0.02**
>50	0.10 (0.01,0.90)	**0.04**	0.09 (0.01,0.58)	**0.01**	0.11 (0.01,0.92)	**0.04**	0.23 (0.06,0.91)	**0.04**
Years of working in the current workplace (years)								
≤5	1.00		1.00		1.00		1.00	
6-10	0.39 (0.13,1.14)	0.09	0.97 (0.28,3.30)	0.96	0.36 (0.16,0.81)	**0.01**	0.76 (0.33,1.77)	0.52
11-15	0.34 (0.09,1.36)	0.13	0.64 (0.18,2.32)	0.50	0.29 (0.08,1.03)	0.06	0.36 (0.12,1.11)	0.07
16-20	0.15 (0.02,1.23)	0.08	0.22 (0.03,1.85)	0.16	0.22 (0.02,2.05)	0.18	0.12 (0.02,1.02)	0.05
>20	0.42 (0.05,3.84)	0.44	0.23 (0.05,1.14)	0.07	NA	NA	0.32 (0.06,1.58)	0.16
Current employment status								
Full-time	1.00		1.00		1.00		1.00	
Part-time	0.60 (0.07,5.06)	0.64	0.26 (0.03,2.05)	0.20	0.37 (0.04,3.21)	0.37	0.40 (0.09,1.85)	0.24
Training status in HKAM (Physician)								
Nil	NA		1.00		NA		NA	
Basic Trainee	1.00		NA		NA	NA	NA	NA
Higher Trainee	0.30 (0.10,1.25)	0.08	NA	NA	NA	NA	NA	NA
Academy Fellow	0.32 (0.19,1.78)	0.09	0.36 (0.13,1.01)	0.05	NA	NA	NA	NA
Job position (Nurse)								
Enrolled nurse	NA	NA	NA	NA	1.00		1.00	
Registered nurse	NA	NA	NA	NA	1.80 (0.82,3.93)	0.14	2.61 (1.20,5.66)	**0.02**
Advanced practice nurse	NA	NA	NA	NA	0.80 (0.24,2.72)	0.72	2.47 (0.73,8.35)	0.15
Type of practice/work setting								
Clinic where physician practice as solo	NA	NA	1.00		NA	NA	1.00	
Clinic where with partners/ group practice	NA	NA	0.94 (0.28,3.12)	0.92	NA	NA	1.02 (0.38,2.73)	0.97
General Outpatient Clinic	1.00		3.08 (0.81,11.70)	0.10	1.00		0.93 (0.30,2.84)	0.89
Family Medicine Integrated/ Specialist Clinic	0.50 (0.16,1.57)	0.23	2.67 (0.65,9.97)	0.17	1.23 (0.53,2.83)	0.63	1.95 (0.64,6.00)	0.24
Drinking in the past year								
Yes	1.00		1.00		1.00		1.00	
No	1.92 (0.78,4.75)	0.16	1.22 (0.48,3.08)	0.68	0.59 (0.28,1.23)	0.16	1.51 (0.72,3.14)	0.27

*AUDIT* Alcohol Use Disorders Identification Test, *BI* Brief intervention, *OR* Crude odds ratios ,*CI* Confidence interval, *NA* Not applicable

Univariate associations between independent variables of interest (CFIR constructs and attitudes toward drinking) and SBI implementation were presented in Supplementary Table 4. Among physicians and nurses in both sectors, participants were more likely to implement SBI when perceiving stronger evidence supporting SBI (AOR: 1.25-1.36, *p*=0.03-0.04), better knowledge (AOR: 1.19-1.61, *p*=0.02-0.04) and self-efficacy (AOR: 1.12-1.18, *p*=0.02-0.04) to implement SBI, more available resources (AOR: 1.17-1.35, *p*=0.003-0.04) and clearer planning for SBI implementation in the clinics (AOR: 1,29-1.59, *p*=0.01-0.03) were reported. Participants in both sectors were less likely to implement SBI when perceiving SBI implementation to be complicated (AOR: 0.70-0.82, *p*=0.01-0.04) and of higher cost (AOR: 0.46-0.61, *p*=0.02-0.04), and drinking approved by Chinese culture (AOR: 0.27-0.50, *p*=0.001-0.02) were reported. Participants were more likely to implement SBI when perceiving SBI fit better with the existing practice in their clinics (adaptability) (AOR: 1.40 & 1.55, *p*=0.02 & 0.008) and better leadership engagement supporting SBI (AOR: 1.91 & 1.75, *p*=0.03 & 0.02) were reported among physicians and nurses in the public sector, but not among those in the private sector. Participants were more likely to implement SBI when perceiving a strong need (AOR: 1.95 & 2.09, *p*=0.01) and greater importance to implement SBI (AOR: 2.08 & 2.17, *p*=0.03 & 0.02) in their clinics were reported among physicians in both public and private sectors, but not among nurses. Participants were more likely to implement SBI when perceiving an organizational culture supporting SBI was reported among nurses in public and private sectors (AOR: 1.31 & 1.22, *p*=0.001 & 0.004), but not among physicians (Table 6).

**Table 6:** Factors associated with the use of SBI (both AUDIT and BI) at least one episode in the past year (*N*=577)

	Physicians				Nurses			
	Public (*N*=137)		Private (*N*=145)		Public (*N*=140)		Private (*N*=155)	
	AOR (95%CI)	*P* values	AOR (95%CI)	*P* values	AOR (95%CI)	*P* values	AOR (95%CI)	*P* values
**Intervention characteristics**								
Evidence Strength Scale	1.30 (1.01,1.67)	**0.04**	1.36 (1.04,1.77)	**0.03**	1.27 (1.01,1.58)	**0.04**	1.25 (1.01,1.55)	**0.04**
Relative advantage	0.99 (0.49,2.00)	0.97	1.16 (0.63,2.14)	0.63	0.90 (0.49,1.66)	0.74	1.23 (0.67,2.27)	0.51
Adaptability Scale	1.40 (1.19,1.63)	**0.02**	1.24 (0.81,1.88)	0.32	1.55 (1.13,2.14)	**0.008**	0.97 (0.71,1.33)	0.84
Complexity Scale	0.70 (0.52,0.96)	**0.01**	0.75 (0.57,0.98)	**0.03**	0.82 (0.69,0.99)	**0.04**	0.76 (0.61,0.96)	**0.03**
Cost	0.50 (0.27,0.94)	**0.03**	0.61 (0.31,0.85)	**0.04**	0.46 (0.28,0.86)	**0.02**	0.55 (0.33,0.92)	**0.03**
**Outer setting**								
Cosmopolitanism	1.19 (0.93,2.26)	0.09	1.08 (0.73,1.60)	0.70	0.94 (0.66,1.34)	0.74	1.11 (0.79,1.55)	0.54
Patient Needs Scale	1.05 (0.85,1.30)	0.63	1.03 (0.85,1.26)	0.74	0.92 (0.75,1.13)	0.43	1.04 (0.87,1.23)	0.70
External policy or incentives	1.35 (0.76,2.41)	0.31	0.77 (0.45,1.31)	0.33	1.57 (0.98,2.52)	0.06	1.06 (0.70,1.62)	0.77
**Inner setting**								
Relative priority	1.95 (1.15,3.31)	**0.01**	2.09 (1.16,3.75)	**0.01**	0.78 (0.51,1.20)	0.26	1.21 (0.79,1.83)	0.41
Tension for change	2.08 (1.09,3.96)	**0.03**	2.17 (1.16,4.05)	**0.02**	1.00 (0.86,1.18)	0.97	1.35 (0.83,2.21)	0.23
Network and communication	1.27 (0.65,2.49)	0.48	0.84 (0.44,1.60)	0.60	0.70 (0.40,1.20)	0.19	1.09 (0.62,1.94)	0.76
Leadership engagement	1.91 (1.06,3.44)	**0.03**	0.63 (0.36,1.10)	0.10	1.75 (1.10,2.81)	**0.02**	1.11 (0.73,1.68)	0.64
Organizational Culture Scale	1.04 (0.92,1.18)	0.51	1.15 (0.88,1.35)	0.11	1.31 (1.08,1.87)	**0.001**	1.22 (1.04,1.77)	**0.004**
Goals and Feedback Scale	0.99 (0.72,1.37)	0.95	0.91 (0.65,1.29)	0.61	1.06 (0.82,1.37)	0.65	0.88 (0.67,1.16)	0.35
Available Resources Scale	1.35 (1.11,1.65)	**0.003**	1.30 (1.06,1.61)	**0.01**	1.29 (1.09,1.54)	**0.004**	1.17 (1.01,1.36)	**0.04**
**Characteristics of individuals**								
Knowledge Scale	1.61 (1.09,2.36)	**0.02**	1.44 (1.02,2.07)	**0.04**	1.22 (1.03,1.44)	**0.02**	1.19 (1.00,1.41)	**0.04**
Positive Beliefs Scale	1.02 (0.80,1.28)	0.90	0.78 (0.59,1.01)	0.06	1.14 (0.91,1.43)	0.24	1.24 (0.98,1.57)	0.07
Negative Beliefs Scale	0.81 (0.61,1.07)	0.14	0.87 (0.67,1.13)	0.29	1.21 (0.93,1.55)	0.15	0.88 (0.70,1.12)	0.31
Self-efficacy Scale	1.18 (1.02,1.37)	**0.03**	1.17 (1.03,1.35)	**0.04**	1.12 (1.01,1.24)	**0.03**	1.16 (1.03,1.31)	**0.02**
**Process**								
Planning Scale	1.59 (1.12,2.27)	**0.01**	1.48 (1.07,2.13)	**0.02**	1.39 (1.06,1.82)	**0.02**	1.29 (1.05,1.61)	**0.03**
Engaging	0.91 (0.54,1.52)	0.71	1.04 (0.64,1.69)	0.87	0.65 (0.42,1.01)	0.06	1.12 (0.70,1.77)	0.64
Executing	0.68 (0.43,1.07)	0.09	1.17 (0.78,1.76)	0.45	1.31 (0.94,1.82)	0.11	0.79 (0.57,1.12)	0.18
Reflecting and Evaluating Scale	1.17 (0.83,1.66)	0.37	0.94 (0.69,1.29)	0.71	1.14 (0.87,1.49)	0.34	0.99 (0.77,1.27)	0.92
**Cultural attitudes toward drinking**								
Social approval of drinking	0.44 (0.22,0.87)	**0.02**	0.27 (0.13,0.57)	**0.001**	0.50 (0.31,0.81)	**0.004**	0.63 (0.38,1.03)	0.06
Functions of drinking in the Chinese Culture Scale	1.10 (0.92,1.32)	0.29	0.88 (0.75,1.04)	0.13	1.02 (0.89,1.16)	0.77	0.93 (0.80,1.08)	0.32
Value of moderation proposed by Confucianism/ Taoism to control drinking	1.33 (0.82,2.16)	0.07	1.31 (0.83,2.08)	0.08	1.25 (0.74,2.26)	0.09	1.27 (0.84,2.28)	0.10
Stigmatization issue of alcohol use	0.79 (0.67,1.68)	0.08	0.85 (0.56,1.95)	0.11	0.82 (0.49,1.37)	0.14	0.76 (0.48,1.20)	0.10

*AUDIT* Alcohol Use Disorders Identification Test, *BI* Brief intervention, *CI* Confidence interval, *AOR* adjusted odds ratios, odds ratios adjusted for significant background characteristics listed in Table 3

## Discussion

This is the first original study investigating facilitators and barriers to implementing SBI among primary care providers in China. We applied the 2009 version of CFIR as the theoretical framework, which provided a comprehensive and standardized list of implementation-related constructs that may be relevant to explain why there is an SBI research-practice gap, between evidence for SBI and its use in routine primary care practice. By integrating qualitative and quantitative methods and data, this study gained both breadth and depth of understanding of SBI implementation in China. Our results suggested that some facilitators and barriers of SBI implementation were different between physicians and nurses, and between healthcare providers in the public and private sectors. The findings have implications for service planning and policymaking.

There was a large gap between SBI implementation and its recommendation in Hong Kong, as only 14.5-16.8% of physicians and 26.5-30.0% of nurses implemented SBI in the past year. Even among primary care providers who have implemented SBI, the coverage of SBI was quite low among their patients. The level of SBI implementation in Hong Kong was worse than that in other countries, such as Sweden (36.1%) [36], the United Kingdom (40.0%) [37], or Canada (75.0%) [38]. There is hence a strong need to improve SBI implementation in primary care settings in Hong Kong.

The facilitators and barriers identified by this study provided some implications to improve SBI implementation. Among CFIR constructs identified in the qualitative part of the study, the quantitative study findings confirmed that perceiving stronger evidence supporting SBI, better knowledge and self-efficacy to implement SBI, more available resources, and clearer planning for SBI implementation in the clinics were common facilitators among both physicians and nurses in both sectors. Such findings were similar to those observed among physicians and nurses in some Western countries [39–42]. Based on the CFIR-ERIC (Expert Recommendations for Implementing Change) tool for matching determinants and strategies to address these determinants [26, 43], some strategies may be useful to enhance these facilitators. These strategies included identifying and preparing champions, conducting educational meetings, and capturing and sharing local knowledge [44]. It is important to identify and prepare some physicians/nurses (champions) who dedicate themselves to allocating useful SBI resources to physicians and nurses, supporting, marketing, and driving through the implementation of SBI, overcoming indifference or resistance that the SBI implementation may provoke in the clinics. Educational meetings with physicians, nurses, as well as administrators should be conducted to share information about SBI (e.g., evidence, content, and progress of implementation) with them. It is also necessary to capture successful cases from implementation sites on how physicians and nurses have made SBI work and share them with other colleagues, which would increase their self-efficacy in implementing SBI.

In line with the findings in the United Kingdom, Slovenia, the United States, New Zealand, and Germany, common barriers applied to four sub-groups included the perceptions that SBI implementation was complicated and of high cost [45–49]. Based on the CFIR-ERIC matching tool, strategies to address these barriers include accessing new funding, promoting adaptability, and developing a formal implementation blueprint [44]. Accessing new funding sources could involve new uses of existing money or accessing block grants for SBI delivery, such as employing new staff to facilitate the implementation. Promoting adaptability is to identify the ways SBI can be tailored to meet local needs and clarify which elements of SBI must be maintained to preserve fidelity. Developing a formal implementation blueprint includes all goals and strategies for implementing SBI. The blueprint should include the aim/purpose of the implementation, timeframe, milestones, and appropriate performance/progress measures. Apart from the CFIR constructs, physicians’ or nurses’ acceptance of drinking was found to be a barrier to implementing SBI. Regular meetings/seminars about up-to-date findings on the harms of alcohol should be organized for physicians and nurses.

Perceiving SBI fit better with the existing practice in their clinics and better leadership engagement supporting SBI were facilitators only in the public sector, but not in the private sector. In the public sector, physicians and nurses have to complete a certain amount of consultation assigned by their supervisors on time. Therefore, they have less flexibility in work arrangements than those working in the private sector. In addition to identifying and preparing champions and promoting adaptability, conducting local consensus discussions is a potentially useful strategy recommended by the CFIR-ERIC matching tool to address these barriers [44]. Managers or supervisors should hold discussions with physicians and nurses that address whether the alcohol problem is crucial among their patients in the clinic and how SBI should be implemented appropriately to address this problem.

In line with our qualitative findings, perceiving a stronger need and greater importance to implement SBI were facilitators only among physicians, but not among nurses. Compared to nurses, physicians usually have less time to communicate with their patients. Perceiving needs and importance of SBI implementation would be crucial factors for physicians’ decision to implement such practice, especially when the patients present multiple health problems. In addition to conducting local consensus discussions, strategies generated from the CFIR-ERIC tool include conducting local needs assessment and assessing for readiness [44]. With updated data about patients’ drinking habits and their diseases related to alcohol consumption, there is a need to assess various aspects of the clinic to determine its degree of readiness to implement, such as whether physicians have sufficient knowledge about SBI, their confidence in using SBI, availability of resources and so on.

Furthermore, perceiving an organizational culture supporting SBI was a unique facilitator in nurses. Research has shown that organizational culture is a key factor to improve nurse performance [50]. Organizational culture includes but is not limited to leadership, cooperation among nurses, organizational structure, systems and rewards, and job design [50]. Developing organizational culture is a recognized instrument tool for improving the work performance of nurses, emphasizing core values necessary for individual and organizational effectiveness [51].

This study had several limitations. First, similar to previous studies targeting physicians and nurses, the response rate was relatively low [52, 53], and we only covered four out of seven clusters of public sectors. Selection bias existed for the recruitment of survey participants. Cautious should be taken when generalizing the findings to primary care providers in Hong Kong. Second, some measurements were self-constructed by this study and were not validated by external studies. However, the reliability of these measurements was acceptable. Third, SBI practice might be over-reported due to social desirability. Fourth, this study was conducted during the COVID-19 pandemic. However, the impact of COVID-19 on SBI implementation may be limited as no informant mentioned it as a barrier in the qualitative study. The qualitative study was conducted from July to December 2021 when COVID-19 was stable and well-controlled in Hong Kong. The number of local infected cases was very low during this period and the services provided in the primary care settings resumed normal. This might explain why no informants in the qualitative study mentioned COVID-19 as a barrier of SBI implementation.Fifth, this was a cross-sectional study and could not establish causal relationships. Sixth, among physicians and nurses who had performed SBI in the past year, they only provided it to 1-25% of their patients. The “1-25%” is a large interval. In the survey, the participants were asked to estimate the proportion of their patients who were asked about their alcohol consumption using the AUDIT or received brief intervention. The participants were provided the following response categories: (i) 0%, (ii) 1-25%, (iii) 26-50%, (iv) 51-75%, (v) >75%. Seventh, the survey results showed that there was still a proportion of people who reported that they did not know SBI very well. Lack of knowledge might affect the validity of their responses related to SBI. Finally, after the completion of our study, a new version of the CFIR was published in October 2022 [54]. The 2022 version included 32 new constructs (e.g., critical incidents in the outer setting, information technology infrastructure in the inner setting, COM-B system in the characteristics of individuals, and adapting in the implementation process) [54]. It was a limitation that we did not include these new constructs in this study. Interestingly, two constructs identified by our qualitative part that could not be covered by the 2009 version could match two new constructs in the updated version of CFIR. Stigmatization of alcohol dependence could be matched to social pressure, and the value of moderation proposed by Confucianism and Taoism in controlling drinking in the Chinese context could be matched to local attitudes.

## Conclusions

There was a significant gap between SBI evidence and SBI implementation in primary care settings in Hong Kong, China. Only 22% of the participants had performed SBI at least one episode in the past year. Evidence strength, knowledge, self-efficacy, available resources, and planning were facilitators of SBI implementation in all sub-groups of participants, whilst cost, complexity, and drinking approved by the Chinese culture were barriers applicable to all participants. Additionally, a few unique facilitators were found for type of sectors and professions, i.e,, adaptability and leadership engagement for the public sector, relative priority and tension for change for physicians, and organizational culture for nurses. Implementation strategies should be developed targeting different groups of healthcare providers in an attempt to improve the implementation of SBI in the future.

### Supplementary Information

**Supplementary Material 1.** 

## Data Availability

The data presented in this study are available from the corresponding author upon request. The data are not publicly available as they contain sensitive personal behaviors.

## References

[1] World Health Organization. Factsheets: Alcohol. Available from: https://www.who.int/news-room/fact-sheets/detail/alcohol.

[2] Griswold MG, Fullman N, Hawley C, Arian N, Zimsen SRM, Tymeson HD (2018). Alcohol use and burden for 195 countries and territories, 1990–2016: a systematic analysis for the Global Burden of Disease Study 2016. Lancet.

[3] Ding LJ, Liang YJ, Tan ECK, Hu Y, Zhang C, Liu YX, et al. Smoking, heavy drinking, physical inactivity, and obesity among middle-aged and older adults in China: cross-sectional findings from the baseline survey of CHARLS 2011-2012. BMC Public Health. 2020;20(1). 10.1186/s12889-020-08625-5 PMC7336642 32631359

[4] Department of Health. Behavioural Risk Factor Survey (April 2016). 2017 Available from: https://www.chp.gov.hk/files/pdf/brfa_report_april_2016_eng.pdf.

[5] Ballesteros J, Duffy JC, Querejeta I, Arino J, Gonzalez-Pinto A (2004). Efficacy of brief interventions for hazardous drinkers in primary care: Systematic review and meta-analyses. Alcohol Clin Exp Res.

[6] Bertholet N, Daeppen JB, Wietlisbach V, Fleming M, Burnand B (2005). Reduction of alcohol consumption by brief alcohol intervention in primary care - Systematic review and meta-analysis. Arch Intern Med.

[7] Kaner EFS, Beyer FR, Muirhead C, Campbell F, Pienaar ED, Bertholet N, et al. Effectiveness of brief alcohol interventions in primary care populations. Cochrane Db Syst Rev. 2018(2). 10.1002/14651858.CD004148.pub4 PMC6491186 29476653

[8] Moyer A, Finney JW, Swearingen CE, Vergun P (2002). Brief interventions for alcohol problems: a meta-analytic review of controlled investigations in treatment-seeking and non-treatment-seeking populations. Addiction.

[9] Angus C, Scafato E, Ghirini S, Torbica A, Ferre F, Struzzo P, et al. Cost-effectiveness of a programme of screening and brief interventions for alcohol in primary care in Italy. BMC Fam Pract. 2014;15. 10.1186/1471-2296-15-26 PMC3936801 24502342

[10] Purshouse RC, Brennan A, Rafia R, Latimer NR, Archer RJ, Angus CR (2013). Modelling the cost-effectiveness of alcohol screening and brief interventions in primary care in England. Alcohol Alcoholism.

[11] Moyer VA, Preventive Services Task F. Screening and behavioral counseling interventions in primary care to reduce alcohol misuse: U.S. preventive services task force recommendation statement. Ann Intern Med. 2013;159(3):210-8. 10.7326/0003-4819-159-3-201308060-00652 23698791

[12] World Health Organization. Global Status Report on Noncommunicable Diseases 2014 [Available from: https://www.who.int/nmh/publications/ncd-status-report-2014/en.

[13] National Institute for Health and Clinical Excellence (NICE). Alcohol-use-disorders: diagnosis, assessment and management of harmful drinking and alcohol dependence. London: National Institute for Health and Clinical Excellence (NICE); 2011. Available from: https://www.nice.org.uk/guidance/cg115. 31886968

[14] Royal Australian College of General Practitioners (RACGP). Smoking, Nutrition, Alcohol and Physical Activity (SNAP)— a population health guide to behavioural risk factors in general practice, 2nd edition. East Melbourne: Royal Australian College of General Practitioners; 2015. Available from: https://www.racgp.org.au/clinical-resources/clinical-guidelines/key-racgp-guidelines/view-all-racgp-guidelines/snap.

[15] Seppanen KK, Aalto M, Seppa K (2012). Institutionalization of brief alcohol intervention in primary health care-the Finnish case. Alcohol Clin Exp Res.

[16] Ockene JK, Adams A, Hurley TG, Wheeler EV, Hebert JR (1999). Brief physician- and nurse practitioner–delivered counseling for high-risk drinkers. Arch Intern Med.

[17] Aalto M, Hyvonen S, Seppa K (2006). Do primary care physicians’ own AUDIT scores predict their use of brief alcohol intervention? A cross-sectional survey. Drug Alcohol Depen.

[18] Israel Y, Hollander O, Sanchez-Craig M, Booker S, Miller V, Gingrich R et al. Screening for problem drinking and counseling by the primary care physician-nurse team. 1996;20(8): 1443-1450. 10.1111/j.1530-0277.1996.tb01147.x 8947323

[19] Costa M, Yaya I, Mora M, Marcellin F, Villotitch A, Berenger C (2019). Barriers and levers in screening and care for alcohol use disorders among French general practitioners: results from a computer-assisted telephone interview-based survey. Alcohol Treat Q.

[20] Chan PSF, Fang Y, Wong MCS, Huang JJ, Wang ZX, Yeoh EK. Using Consolidated Framework for Implementation Research to investigate facilitators and barriers of implementing alcohol screening and brief intervention among primary care health professionals: a systematic review. Implement Sci. 2021;16(1). 10.1186/s13012-021-01170-8 PMC8605518 34801041

[21] Hong Kong Government. Overview of the Health Care System in Hong Kong. Available from: https://www.gov.hk/en/residents/health/hosp/overview.htm.

[22] Hong Kong Department of Health. Useful Information. Available from: https://www.dh.gov.hk/english/useful/useful_cme/useful_cmeni2310.html.

[23] Hong Kong Department of Health. Alcohol Fails Toolkits [Available from: https://www.change4health.gov.hk/en/alcoholfails/toolkits.html.

[24] Keith RE, Crosson JC, O'Malley AS, Cromp D, Taylor EF. Using the Consolidated Framework for Implementation Research (CFIR) to produce actionable findings: a rapid-cycle evaluation approach to improving implementation. Implement Sci. 2017;12. 10.1186/s13012-017-0550-7 PMC5303301 28187747

[25] Damschroder LJ, Aron DC, Keith RE, Kirsh SR, Alexander JA, Lowery JC. Fostering implementation of health services research findings into practice: a consolidated framework for advancing implementation science. Implement Sci. 2009;4. 10.1186/1748-5908-4-50 PMC2736161 19664226

[26] Consolidated Framework for Implementation Research: Strategy design. Available from: https://cfirguide.org/choosing-strategies.

[27] Hsieh HF, Shannon SE (2005). Three approaches to qualitative content analysis. Qual Health Res.

[28] Assarroudi A, Nabavi FH, Armat MR, Ebadi A, Vaismoradi M (2018). Directed qualitative content analysis: the description and elaboration of its underpinning methods and data analysis process. J Res Nurs.

[29] Wei H, Watson J (2019). Healthcare interprofessional team members' perspectives on human caring: a directed content analysis study. Int J Nurs Sci.

[30] Sabzmakan L, Morowatisharifabad MA, Mohammadi E, Mazloomy-Mahmoodabad SS, Rabiei K, Naseri MH (2014). Behavioral determinants of cardiovascular diseases risk factors: a qualitative directed content analysis. Arya Atheroscler.

[31] Low LL, Ab Rahim FI, Johari MZ, Abdullah Z, Aziz SHA, Suhaimi NA, et al. Assessing receptiveness to change among primary healthcare providers by adopting the consolidated framework for implementation research (CFIR). BMC Health Serv Res. 2019;19. 10.1186/s12913-019-4312-x PMC6636000 31311538

[32] Hong Kong Hospital Authority. Clusters, Hospitals & Institutions. Available from: https://www.ha.org.hk/visitor/template101.asp.

[33] Hong Kong Health Bureau. Primary Care Directory. Available from: https://apps.pcdirectory.gov.hk/Public/TC/AdvancedSearch?ProfID=RMP.

[34] Chang J, Shrake E, Rhee S. Patterns of alcohol use and attitudes toward drinking among Chinese and Korean American college students. J Ethn Subst Abuse. 2008;7(3):341-56. Keyword Heading Alcohol use Attitudes toward drinking Chinese and Korean college student. 10.1080/15332640802313346 19042813

[35] King M (1986). At risk drinking among general-practice attenders - validation of the cage questionnaire. Psychol Med.

[36] Holmqvist M, Bendtsen P, Spak F, Rommelsjo A, Geirsson M, Nilsen P (2008). Asking patients about their drinking - A national survey among primary health care physicians and nurses in Sweden. Addict Behav.

[37] Wilson GB, Lock CA, Heather N, Cassidy P, Christie MM, Kaner EF (2011). Intervention against Excessive Alcohol Consumption in Primary Health Care: A Survey of GPs' Attitudes and Practices in England 10 Years On. Alcohol Alcoholism.

[38] Rush B, Bass M, Stewart M, Mccracken E, Labreque M, Bondy S (1994). Detecting, preventing, and managing patients alcohol-problems. Can Fam Phys.

[39] McAvoy BR, Donovan RJ, Jalleh G, Saunders JB, Wutzke SE, Lee N, et al. General practitioners, prevention and alcohol-a powerful cocktail? Facilitators and inhibitors of practising preventive medicine in general and early intervention for alcohol in particular: A 12-nation key informant and general practitioner study. Drugs. 2001;.8(2).

[40] Geirsson M, Bendtsen P, Spak F (2005). Attitudes of Swedish general practitioners and nurses to working with lifestyle change, with special reference to alcohol consumption. Alcohol Alcoholism.

[41] Miquel L, Lopez-Pelayo H, Nuno L, Arbesu JA, Zarco J, Manthey J (2018). Barriers to implement screening for alcohol consumption in Spanish hypertensive patients. Fam Pract.

[42] Friedmann PD, McCullough D, Chin MH, Saitz R (2000). Screening and intervention for alcohol problems - A national survey of primary care physicians and psychiatrists. J Gen Intern Med.

[43] Waltz TJ, Powell BJ, Fernandez ME, Abadie B, Damschroder LJ. Choosing implementation strategies to address contextual barriers: diversity in recommendations and future directions. Implement Sci. 2019;14. 10.1186/s13012-019-0892-4 PMC6489173 31036028

[44] Powell BJ, Waltz TJ, Chinman MJ, Damschroder LJ, Smith JL, Matthieu MM, et al. A refined compilation of implementation strategies: results from the Expert Recommendations for Implementing Change (ERIC) project. Implement Sci. 2015;10. 10.1186/s13012-015-0209-1 PMC4328074 25889199

[45] Rapley T, May C, Frances Kaner E (2006). Still a difficult business? Negotiating alcohol-related problems in general practice consultations. Soc Sci Med.

[46] Susic T, J K, M K, Email Susic T, tonka, poplas sm, et al. Why do general practitioners not screen and intervene regarding alcohol consumption in Slovenia? A focus group study. 2010. 10.1007/s00508-010-1335-z 20517676

[47] Miller PM, Stockdell R, Nemeth  L, Feifer C, Jenkins RG, Liszka H, Ornstein S, Nietert PJ (2006). Initial steps taken by nine primary care practices to implement alcohol screening guidelines with hypertensive patients: The AA-TRIP project.

[48] Mules T, Taylor J, Price R, Walker L, Singh B, Newsam P (2012). Addressing patient alcohol use: a view from general practice. J Prim Health Care.

[49] Kraus L, Schulte B, Manthey J, Rehm J (2017). Alcohol screening and alcohol interventions among patients with hypertension in primary health care: an empirical survey of German general practitioners. Addict Res Theory.

[50] Ekasari PA, Noermijati N, Dewanto A (2020). Organizational culture: a key factor to improve nurse performance. Enfermería Clínica.

[51] Banaszak-Holl J, Castle NG, Lin MK, Shrivastwa N, Spreitzer G (2013). The Role of Organizational Culture in Retaining Nursing Workforce. Gerontol.

[52] Fang Y, Wang HH, Liang M, Yeung MS, Leung C, Chan CH, et al. The adoption of hypertension reference framework: An investigation among primary care physicians of Hong Kong. 2018. 10.1371/journal.pone.0205529 PMC6177174 30300397

[53] Wong MC, Wang HH, Kwan MW, Chan WM, Fan CK, Liang M,, et al. The adoption of the Reference Framework for diabetes care among primary care physicians in primary care settings: A cross-sectional study. 2016. 10.1097/MD.0000000000004108 PMC4979772 27495018

[54] Damschroder LJ, Reardon CM, Widerquist MAO, Lowery J. The updated Consolidated Framework for Implementation Research based on user feedback. Implement Sci. 2022;17(75). 10.1186/s13012-022-01245-0 PMC9617234 36309746

