# Supplementary material for: Applying the Consolidated Framework for Implementation Research to investigate factors of implementing alcohol screening and brief intervention among primary care physicians and nurses in Hong Kong, China: an exploratory sequential mixed-method study
Source: Implement Sci Commun. 2024 May 6;5:52. doi: 10.1186/s43058-024-00590-z (PMC11071187; doi:10.1186/s43058-024-00590-z)
Supplement: Supplementary file 1 — Supplementary Material 1. [file 43058_2024_590_MOESM1_ESM.docx]

Supplementary Table 1 Measurements based on the CFIR used in the quantitative study

| Domains/constructs | Name of the measurement | Adapted from validated tools or self-constructed | Name of the original tools | Number of items | Cronbach’s alpha |
| --- | --- | --- | --- | --- | --- |
| **Intervention characteristics** |  |  |  |  |  |
| Evidence Strength | Evidence Strength Scale | Adapted from validated tools | Organizational Readiness to Change Assessment | 3 | 0.85 |
| Relative advantage | Relative advantage | Adapted from validated tools | Assessment of the Promotion of Colorectal Cancer Screening | 1 | NA |
| Adaptability Scale | Adaptability Scale | Adapted from validated tools | Assessment of the Promotion of Colorectal Cancer Screening | 2 | 0.79 |
| Complexity | Complexity Scale | Adapted from validated tools | Assessment of the Promotion of Colorectal Cancer Screening | 4 | 0.78 |
| Cost | Cost | Adapted from validated tools | Asking Patients about Alcohol Consumption Scale | 1 | NA |
| **Outer setting** |  |  |  |  |  |
| Cosmopolitanism | Cosmopolitanism | Adapted from validated tools | Experiences in Delivering Screening and Advice for Alcohol Scale | 1 | NA |
| Patient Needs | Patient Needs Scale | Adapted from validated tools | Asking Patients about Alcohol Consumption Scale | 5 | 0.76 |
| External policy or incentives | External policy or incentives | Adapted from validated tools | Perception of Factors Influencing Delivery of Intervention Scale | 1 | NA |
| **Inner setting** |  |  |  |  |  |
| Relative priority | Relative priority | Self-constructed | NA | 1 | NA |
| Tension for change | Tension for change | Self-constructed | NA | 1 | NA |
| Network and communication | Network and communication | Adapted from validated tools | Asking Patients about Alcohol Consumption Scale | 1 | NA |
| Leadership engagement | Leadership engagement | Adapted from validated tools | Asking Patients about Alcohol Consumption Scale | 1 | NA |
| Organizational Culture | Organizational Culture Scale | Adapted from validated tools | Organizational Readiness to Change Assessment | 4 | 0.85 |
| Goals and Feedback | Goals and Feedback Scale | Adapted from validated tools | Assessment of the Promotion of Colorectal Cancer Screening | 2 | 0.76 |
| Available Resources | Available Resources Scale | Adapted from validated tools | Perception of Factors Influencing Delivery of Intervention Scale | 4 | 0.85 |
| **Characteristics of individuals** |  |  |  |  |  |
| Knowledge | Knowledge Scale | Adapted from validated tools | The Short Alcohol and Alcohol Problems Perception Scale | 2 | 0.82 |
| Positive Beliefs | Positive Beliefs Scale | Adapted from validated tools | The Short Alcohol and Alcohol Problems Perception Scale | 4 | 0.65 |
| Negative Beliefs | Negative Beliefs Scale | Adapted from validated tools | The Short Alcohol and Alcohol Problems Perception Scale | 3 | 0.74 |
| Self-efficacy | Self-efficacy Scale | Adapted from validated tools | Experiences in Delivering Screening and Advice for Alcohol Scale | 6 | 0.68 |
| **Process** |  |  |  |  |  |
| Planning | Planning Scale | Adapted from validated tools | Organizational Readiness to Change Assessment | 2 | 0.83 |
| Engaging | Engaging Scale | Adapted from validated tools | Assessment of the Promotion of Colorectal Cancer Screening | 2 | 0.78 |
| Executing | Executing | Adapted from validated tools | Assessment of the Promotion of Colorectal Cancer Screening | 1 | NA |
| Reflecting and Evaluating | Reflecting and Evaluating Scale | Adapted from validated tools | Assessment of the Promotion of Colorectal Cancer Screening | 2 | 0.81 |

NA: not applicable

Supplementary table 2 Frequency distributions of the measurements (N=577)

|  | Physicians | | Nurses | |
| --- | --- | --- | --- | --- |
|  | Public  (N=137) | Private  (N=145) | Public  (N=140) | Private  (N=155) |
|  | N (%) | N (%) | N (%) | N (%) |
| Intervention characteristics |  |  |  |  |
| Evidence Strength Scale |  |  |  |  |
| SBI is supported by randomized controlled trials or other scientific evidence |  |  |  |  |
| Strongly disagree | 0 | 2 (1.4) | 3 (2.1) | 2 (1.3) |
| Disagree | 0 | 0 | 6 (4.3) | 13 (8.4) |
| Neutral | 42 (30.7) | 58 (40.0) | 80 (57.1) | 83 (53.5) |
| Agree | 88 (64.2) | 81 (55.9) | 51 (36.4) | 53 (34.2) |
| Strongly agree | 7 (5.1) | 4 (2.8) | 0 | 4 (2.6) |
| SBI conforms to the opinions of clinical experts |  |  |  |  |
| Strongly disagree | 0 | 2 (1.4) | 1 (0.7) | 4 (2.6) |
| Disagree | 0 | 2 (1.4) | 5 (3.6) | 6 (3.9) |
| Neutral | 52 (38.0) | 52 (35.9) | 77 (55.0) | 77 (49.7) |
| Agree | 78 (56.9) | 85 (58.6) | 57 (40.7) | 64 (41.3) |
| Strongly agree | 7 (5.1) | 4 (2.8) | 0 | 4 (2.6) |
| SBI is supported by clinical experience with patients in primary care settings |  |  |  |  |
| Strongly disagree | 3 (2.2) | 1 (0.7) | 2 (1.4) | 5 (3.2) |
| Disagree | 3 (2.2) | 5 (3.4) | 8 (5.7) | 6 (3.9) |
| Neutral | 46 (33.6) | 59 (40.7) | 71 (50.7) | 70 (45.2) |
| Agree | 78 (56.9) | 74 (51.0) | 58 (41.4) | 68 (43.9) |
| Strongly agree | 7 (5.1) | 6 (4.1) | 1 (0.7) | 6 (3.9) |
| Relative advantage |  |  |  |  |
| Using SBI is more effective than your own prior practices for helping patients reduce alcohol consumption |  |  |  |  |
| Strongly disagree | 0 | 2 (1.4) | 1 (0.7) | 2 (1.3) |
| Disagree | 0 | 4 (2.8) | 9 (6.4) | 14 (9.0) |
| Neutral | 78 (56.9) | 79 (54.5) | 76 (54.3) | 67 (43.2) |
| Agree | 52 (38.0) | 52 (35.9) | 50 (35.7) | 70 (45.2) |
| Strongly agree | 7 (5.1) | 8 (5.5) | 4 (2.9) | 2 (1.3) |
| Adaptability Scale |  |  |  |  |
| Using SBI to help patients reduce alcohol consumption is compatible with current activities /practices in the clinic |  |  |  |  |
| Strongly disagree | 0 | 0 | 3 (2.1) | 0 |
| Disagree | 36 (26.3) | 15 (10.3) | 10 (7.1) | 7 (4.5) |
| Neutral | 55 (40.1) | 50 (34.5) | 72 (51.4) | 57 (36.8) |
| Agree | 40 (29.2) | 51 (35.2) | 52 (37.1) | 72 (46.5) |
| Strongly agree | 6 (4.4) | 29 (20.0) | 3 (2.1) | 19 (12.3) |
| Using SBI to help patients reduce alcohol consumption fits well with the way you like to work |  |  |  |  |
| Strongly disagree | 0 | 0 | 1 (0.7) | 2 (1.3) |
| Disagree | 40 (29.2) | 26 (17.9) | 8 (5.7) | 8 (5.2) |
| Neutral | 57 (41.6) | 65 (44.8) | 79 (56.4) | 53 (34.2) |
| Agree | 37 (27.0) | 28 (19.3) | 45 (32.1) | 72 (46.5) |
| Strongly agree | 3 (2.2) | 26 (17.9) | 7 (5.0) | 20 (12.9) |
| Complexity Scale |  |  |  |  |
| It is difficult to train providers and staff to implement SBI |  |  |  |  |
| Strongly disagree | 1 (0.7) | 0 | 3 (2.1) | 1 (0.6) |
| Disagree | 47 (34.3) | 33 (22.8) | 19 (13.6) | 24 (15.5) |
| Neutral | 49 (35.8) | 64 (44.1) | 68 (48.6) | 74 (47.7) |
| Agree | 33 (24.1) | 42 (29.0) | 47 (33.6) | 52 (33.5) |
| Strongly agree | 7 (5.1) | 6 (4.1) | 3 (2.1) | 4 (2.6) |
| Overall, you believe that it is complicated to implement SBI |  |  |  |  |
| Strongly disagree | 3 (2.2) | 0 | 3 (2.1) | 5 (3.2) |
| Disagree | 13 (9.5) | 27 (18.6) | 14 (10.0) | 22 (14.2) |
| Neutral | 65 (47.4) | 60 (41.4) | 79 (56.4) | 65 (41.9) |
| Agree | 49 (35.8) | 54 (37.2) | 43 (30.7) | 57 (36.8) |
| Strongly agree | 7 (5.1) | 4 (2.9) | 1 (0.7) | 6 (3.9) |
| Using SBI required your clinic to make substantial changes to your previous practice |  |  |  |  |
| Strongly disagree | 0 | 0 | 2 (1.4) | 2 (1.3) |
| Disagree | 13 (9.5) | 37 (25.5) | 6 (4.3) | 7 (4.5) |
| Neutral | 52 (38.0) | 54 (37.2) | 71 (50.7) | 74 (47.7) |
| Agree | 65 (47.4) | 52 (35.9) | 58 (41.4) | 63 (40.6) |
| Strongly agree | 7 (5.1) | 2 (1.4) | 3 (2.1) | 9 (5.8) |
| SBI requires more work than can be done with current resources |  |  |  |  |
| Strongly disagree | 0 | 2 (1.4) | 2 (1.4) | 2 (1.3) |
| Disagree | 16 (11.7) | 14 (9.7) | 7 (5.0) | 9 (5.8) |
| Neutral | 50 (36.5) | 36 (24.8) | 63 (45.0) | 75 (48.4) |
| Agree | 61 (44.5) | 85 (58.6) | 61 (43.6) | 65 (41.9) |
| Strongly agree | 10 (7.3) | 8 (5.5) | 7 (5.0) | 4 (2.6) |
| Cost |  |  |  |  |
| It makes the consultation too long |  |  |  |  |
| Strongly disagree | 0 | 8 (5.5) | 1 (0.7) | 4 (2.6) |
| Disagree | 11 (8.0) | 24 (16.6) | 12 (8.6) | 27 (17.4) |
| Neutral | 23 (16.8) | 42 (29.0) | 26 (18.6) | 56 (36.1) |
| Agree | 69 (50.4) | 50 (34.5) | 68 (48.6) | 51 (32.9) |
| Strongly agree | 34 (24.8) | 21 (14.5) | 33 (23.6) | 17 (11.0) |
| Outer setting |  |  |  |  |
| Cosmopolitanism |  |  |  |  |
| Referring patients to an appropriate service in case of severe problems with alcohol is easy |  |  |  |  |
| Strongly disagree | 2 (1.5) | 0 | 6 (4.3) | 6 (3.9) |
| Disagree | 33 (24.1) | 39 (26.9) | 33 (23.6) | 22 (14.2) |
| Neutral | 49 (35.8) | 50 (34.5) | 68 (48.6) | 70 (45.2) |
| Agree | 46 (33.6) | 50 (34.5) | 31 (22.1) | 48 (31.0) |
| Strongly agree | 7 (5.1) | 6 (4.1) | 2 (1.4) | 9 (5.8) |
| Patient Needs Scale |  |  |  |  |
| It improves your relationship with your patients |  |  |  |  |
| Strongly disagree | 3 (2.2) | 2 (1.4) | 7 (5.0) | 5 (3.2) |
| Disagree | 29 (21.2) | 19 (13.1) | 25 (17.9) | 37 (23.9) |
| Neutral | 59 (43.1) | 81 (55.9) | 85 (60.7) | 74 (47.7) |
| Agree | 46 (33.6) | 39 (26.9) | 22 (15.7) | 35 (22.6) |
| Strongly agree | 0 | 4 (2.8) | 1 (0.7) | 4 (2.6) |
| It makes your patients uneasy |  |  |  |  |
| Strongly disagree | 0 | 4 (2.8) | 6 (4.3) | 3 (1.9) |
| Disagree | 49 (35.8) | 46 (31.7) | 18 (12.9) | 31 (20.0) |
| Neutral | 39 (28.5) | 52 (35.9) | 71 (50.7) | 65 (41.9) |
| Agree | 46 (33.6) | 39 (26.8) | 44 (31.4) | 52 (33.5) |
| Strongly agree | 3 (2.2) | 4 (2.8) | 1 (0.7) | 4 (2.6) |
| You provide better care to your patients |  |  |  |  |
| Strongly disagree | 0 | 0 | 0 | 1 (0.6) |
| Disagree | 0 | 18 | 11 (7.9) | 26 (16.8) |
| Neutral | 7 (5.1) | 32 (17.2) | 49 (35.0) | 50 (32.3) |
| Agree | 127 (92.7) | 79 (71.7) | 76 (54.3) | 65 (41.9) |
| Strongly agree | 3 (2.2) | 16 (11.0) | 4 (2.9) | 13 (8.4) |
| It helps your patients drink less |  |  |  |  |
| Strongly disagree | 0 | 0 | 7 (5.0) | 4 (2.6) |
| Disagree | 26 (19.0) | 25 (17.2) | 29 (20.7) | 33 (21.3) |
| Neutral | 42 (30.7) | 82 (45.5) | 61 (43.6) | 68 (43.9) |
| Agree | 69 (50.4) | 36 (35.9) | 42 (30.0) | 50 (32.3) |
| Strongly agree | 0 | 2 (1.4) | 1 (0.7) | 0 |
| Your patients believe that you should ask your patients how much alcohol they drink |  |  |  |  |
| Strongly disagree | 0 | 5 (3.4) | 1 (0.7) | 2 (1.3) |
| Disagree | 42 (30.7) | 37 (25.5) | 17 (12.1) | 20 (12.9) |
| Neutral | 59 (43.1) | 70 (48.3) | 71 (50.7) | 61 (39.4) |
| Agree | 36 (26.3) | 31 (21.4) | 50 (35.7) | 68 (43.9) |
| Strongly agree | 0 | 2 (1.4) | 1 (0.7) | 4 (2.6) |
| External policy or incentives |  |  |  |  |
| Government health policies in general do not support doctors, nurses and other health care workers who want to practice preventive medicine |  |  |  |  |
| Strongly disagree | 32 (23.4) | 15 (10.3) | 11 (7.9) | 14 (9.0) |
| Disagree | 45 (32.8) | 41 (28.3) | 42 (30.0) | 52 (33.5) |
| Neutral | 39 (28.5) | 56 (38.6) | 60 (42.9) | 59 (38.1) |
| Agree | 13 (9.5) | 23 (15.9) | 15 (10.7) | 20 (12.9) |
| Strongly agree | 8 (5.8) | 10 (6.9) | 12 (8.6) | 10 (6.5) |
| Inner setting |  |  |  |  |
| Relative priority |  |  |  |  |
| Addressing alcohol issue ranks high given other health problems of your patients |  |  |  |  |
| Strongly disagree | 12 (8.8) | 3 (2.1) | 9 (6.4) | 10 (6.5) |
| Disagree | 63 (46.0) | 74 (51.0) | 53 (37.9) | 64 (41.3) |
| Neutral | 23 (16.8) | 48 (33.1) | 65 (46.4) | 62 (40.0) |
| Agree | 36 (26.3) | 16 (11.0) | 10 (7.1) | 13 (8.4) |
| Strongly agree | 3 (2.2) | 4 (2.8) | 3 (2.1) | 6 (3.9) |
| Tension for change |  |  |  |  |
| Alcohol drinking is a critical issue for patients in your clinic |  |  |  |  |
| Strongly disagree | 7 (5.1) | 14 (9.7) | 10 (7.1) | 8 (5.2) |
| Disagree | 43 (31.4) | 77 (53.1) | 30 (21.4) | 45 (29.0) |
| Neutral | 65 (47.4) | 42 (29.0) | 57 (40.7) | 65 (41.9) |
| Agree | 13 (9.5) | 8 (5.5) | 43 (30.7) | 33 (21.3) |
| Strongly agree | 9 (6.6) | 4 (2.8) | 0 | 4 (2.6) |
| Network and communication |  |  |  |  |
| Your colleagues believe that you should ask your patients how much alcohol they drink |  |  |  |  |
| Strongly disagree | 0 | 5 (3.4) | 1 (0.7) | 4 (2.6) |
| Disagree | 19 (13.9) | 21 (14.5) | 17 (12.1) | 17 (11.0) |
| Neutral | 59 (43.1) | 71 (49.0) | 62 (44.3) | 67 (43.2) |
| Agree | 59 (43.1) | 46 (31.7) | 57 (40.7) | 61 (39.4) |
| Strongly agree | 0 | 2 (1.4) | 3 (2.1) | 6 (3.9) |
| Leadership engagement |  |  |  |  |
| Your supervisors/ managers believe that you should ask your patients how much alcohol they drink |  |  |  |  |
| Strongly disagree | 1 (0.7) | 9 (6.2) | 2 (1.4) | 4 (2.6) |
| Disagree | 20 (14.6) | 22 (15.2) | 12 (8.6) | 20 (12.9) |
| Neutral | 47 (34.3) | 70 (48.3) | 53 (37.9) | 59 (38.1) |
| Agree | 66 (48.2) | 42 (29.0) | 69 (49.3) | 63 (40.6) |
| Strongly agree | 3 (2.2) | 2 (1.4) | 4 (2.9) | 9 (5.8) |
| Organizational Culture Scale |  |  |  |  |
| The staff have a sense of personal responsibility for improving patient care and outcomes |  |  |  |  |
| Strongly disagree | 0 | 2 (1.3) | 0 | 2 (1.4) |
| Disagree | 8 (5.7) | 13 (8.4) | 10 (7.3) | 12 (8.3) |
| Neutral | 54 (38.6) | 68 (43.9) | 29 (21.2) | 40 (27.6) |
| Agree | 74 (52.9) | 61 (39.4) | 85 (62.0) | 81 (55.9) |
| Strongly agree | 4 (2.9) | 11 (7.1) | 13 (9.5) | 10 (6.9) |
| The staff cooperate to maintain and improve effectiveness of patient care |  |  |  |  |
| Strongly disagree | 0 | 2 (1.3) | 0 | 0 |
| Disagree | 1 (0.7) | 7 (4.5) | 6 (4.4) | 7 (4.8) |
| Neutral | 47 (33.6) | 52 (33.5) | 10 (7.3) | 41 (28.3) |
| Agree | 85 (60.7) | 83 (53.5) | 111 (81.0) | 85 (58.6) |
| Strongly agree | 7 (5.0) | 11 (7.1) | 10 (7.3) | 12 (8.3) |
| The staff are willing to innovate and/or experiment to improve clinical procedures |  |  |  |  |
| Strongly disagree | 0 | 1 (0.6) | 0 | 2 (1.4) |
| Disagree | 7 (5.0) | 13 (8.4) | 16 (11.7) | 17 (11.7) |
| Neutral | 53 (37.9) | 66 (42.6) | 36 (26.3) | 68 (46.9) |
| Agree | 74 (52.9) | 66 (42.6) | 82 (59.9) | 50 (34.5) |
| Strongly agree | 6 (4.3) | 9 (5.8) | 3 (2.2) | 8 (5.5) |
| The staff are receptive to changes in clinical processes |  |  |  |  |
| Strongly disagree | 0 | 0 | 0 | 2 (1.4) |
| Disagree | 7 (5.0) | 20 (12.9) | 7 (5.1) | 8 (5.5) |
| Neutral | 53 (37.9) | 65 (41.9) | 39 (28.5) | 65 (44.8) |
| Agree | 76 (54.3) | 66 (42.6) | 88 (64.2) | 62 (42.8) |
| Strongly agree | 4 (2.9) | 4 (2.6) | 3 (2.2) | 8 (5.5) |
| Goals and Feedback Scale |  |  |  |  |
| Clinic leaders establish clear goals for SBI |  |  |  |  |
| Strongly disagree | 35 (25.5) | 38 (26.2) | 38 (27.1) | 32 (20.6) |
| Disagree | 37 (27.0) | 40 (27.6) | 30 (21.4) | 45 (29.0) |
| Neutral | 31 (22.6) | 39 (26.9) | 40 (28.6) | 43 (27.7) |
| Agree | 34 (24.8) | 28 (19.3) | 32 (22.9) | 35 (22.6) |
| Strongly agree | 0 | 0 | 0 | 0 |
| Clinic leaders hold staff members accountable for achieving results of SBI |  |  |  |  |
| Strongly disagree | 36 (26.3) | 42 (29.0) | 35 (25.0) | 29 (18.7) |
| Disagree | 30 (21.9) | 35 (24.1) | 32 (22.9) | 45 (27.1) |
| Neutral | 32 (23.4) | 34 (23.4) | 41 (29.3) | 46 (31.6) |
| Agree | 39 (28.5) | 34 (23.4) | 32 (22.9) | 35 (22.6) |
| Strongly agree | 0 | 0 | 0 | 0 |
| Available Resources Scale |  |  |  |  |
| Doctors, nurses and other health care providers have quick and easy counselling materials available |  |  |  |  |
| Strongly disagree | 23 (16.8) | 35 (24.1) | 21 (15.0) | 38 (24.5) |
| Disagree | 28 (20.4) | 59 (40.7) | 40 (28.6) | 45 (29.0) |
| Neutral | 52 (38.0) | 21 (14.5) | 44 (31.4) | 40 (25.8) |
| Agree | 27 (19.7) | 22 (15.2) | 25 (17.9) | 26 (16.8) |
| Strongly agree | 7 (5.1) | 8 (5.5) | 10 (7.1) | 6 (3.9) |
| Doctors, nurses and other health care providers have been trained in counselling for reducing alcohol consumption |  |  |  |  |
| Strongly disagree | 22 (16.1) | 53 (36.6) | 26 (18.6) | 48 (31.0) |
| Disagree | 55 (40.1) | 52 (35.9) | 32 (22.9) | 51 (32.9) |
| Neutral | 33 (24.1) | 13 (9.0) | 45 (32.1) | 37 (23.9) |
| Agree | 17 (12.4) | 20 (13.8) | 26 (18.6) | 15 (9.7) |
| Strongly agree | 10 (7.3) | 7 (4.8) | 11 (7.9) | 4 (2.6) |
| Doctors, nurses and other health care providers have quick and easy screening instruments available |  |  |  |  |
| Strongly disagree | 26 (19.0) | 47 (32.4) | 22 (15.7) | 44 (28.4) |
| Disagree | 34 (24.8) | 53 (36.6) | 44 (31.4) | 49 (31.6) |
| Neutral | 42 (30.7) | 16 (11.0) | 40 (28.6) | 37 (23.9) |
| Agree | 25 (18.2) | 21 (14.5) | 26 (18.6) | 19 (12.3) |
| Strongly agree | 10 (7.3) | 8 (5.5) | 8 (5.7) | 6 (3.9) |
| Support services are readily available for referral of patients with heavy drinking |  |  |  |  |
| Strongly disagree | 19 (13.9) | 33 (22.8) | 13 (9.3) | 39 (25.2) |
| Disagree | 39 (28.5) | 57 (39.3) | 47 (33.6) | 48 (44.0) |
| Neutral | 49 (35.8) | 21 (14.5) | 44 (31.4) | 42 (31.0) |
| Agree | 19 (13.9) | 25 (17.2) | 28 (20.0) | 21 (13.5) |
| Strongly agree | 11 (8.0) | 9 (6.2) | 9 (6.4) | 5 (3.2) |
| Characteristics of individuals |  |  |  |  |
| Knowledge Scale |  |  |  |  |
| You feel you know enough about causes of drinking problems to carry out your role when working with drinkers |  |  |  |  |
| Strongly disagree | 0 | 2 (1.4) | 0 | 2 (1.3) |
| Disagree | 32 (23.4) | 37 (25.5) | 28 (20.0) | 31 (20.0) |
| Neutral | 39 (28.5) | 53 (36.6) | 68 (48.6) | 72 (46.5) |
| Agree | 51 (37.2) | 41 (28.3) | 38 (27.1) | 46 (29.7) |
| Strongly agree | 15 (10.9) | 12 (8.3) | 6 (4.3) | 4 (2.6) |
| In general, you feel you know enough about alcohol screening and brief intervention |  |  |  |  |
| Strongly disagree | 5 (3.6) | 2 (1.4) | 1 (0.7) | 4 (2.6) |
| Disagree | 27 (19.7) | 34 (23.4) | 22 (15.7) | 27 (17.4) |
| Neutral | 41 (29.9) | 46 (31.7) | 63 (45.0) | 60 (38.7) |
| Agree | 50 (36.5) | 46 (31.7) | 51 (36.4) | 56 (36.1) |
| Strongly agree | 14 (10.2) | 17 (11.7) | 3 (2.1) | 8 (5.2) |
| Positive Beliefs Scale ^h^ |  |  |  |  |
| You feel you have the right to ask patients questions about their drinking when necessary |  |  |  |  |
| Strongly disagree | 0 | 0 | 0 | 3 (1.9) |
| Disagree | 3 (2.2) | 7 (4.8) | 8 (5.7) | 7 (4.5) |
| Neutral | 16 (11.7) | 23 (15.9) | 50 (35.7) | 57 (36.8) |
| Agree | 95 (69.3) | 99 (68.3) | 74 (52.9) | 79 (51.0) |
| Strongly agree | 23 (16.8) | 16 (11.0) | 8 (5.7) | 9 (5.8) |
| You feel that your patients believe you have the right to ask them questions about drinking when necessary |  |  |  |  |
| Strongly disagree | 2 (1.5) | 0 | 2 (1.4) | 0 |
| Disagree | 7 (5.1) | 18 (12.4) | 12 (8.6) | 11 (7.1) |
| Neutral | 33 (24.1) | 31 (21.4) | 54 (38.6) | 68 (43.9) |
| Agree | 85 (62.0) | 83 (57.2) | 71 (50.7) | 70 (45.2) |
| Strongly agree | 10 (7.3) | 13 (9.0) | 1 (0.7) | 6 (3.9) |
| In general, it is rewarding to work with drinkers |  |  |  |  |
| Strongly disagree | 0 | 4 (2.8) | 1 (0.7) | 1 (0.6) |
| Disagree | 20 (14.6) | 25 (17.2) | 18 (12.9) | 33 (21.3) |
| Neutral | 78 (56.9) | 85 (58.6) | 86 (61.4) | 78(50.3) |
| Agree | 39 (28.5) | 29 (20.0) | 32 (22.9) | 41 (26.5) |
| Strongly agree | 0 | 2 (1.4) | 3 (2.1) | 2 (1.3) |
| In general, you like drinkers |  |  |  |  |
| Strongly disagree | 4 (2.9) | 8 (5.5) | 15 (10.7) | 10 (6.5) |
| Disagree | 42 (30.7) | 42 (29.0) | 49 (35.0) | 33 (21.3) |
| Neutral | 88 (64.2) | 81(55.9) | 68 (48.6) | 81 (52.3) |
| Agree | 3 (2.2) | 12 (8.3) | 8 (5.7) | 29 (18.7) |
| Strongly agree | 0 | 2 (1.4) | 0 | 2 (1.3) |
| Negative Beliefs Scale |  |  |  |  |
| You feel you do not have much to be proud of when working with drinkers |  |  |  |  |
| Strongly disagree | 0 | 4 (2.8) | 1 (0.7) | 3 (1.9) |
| Disagree | 27 (19.7) | 19 (13.1) | 14 (10.0) | 18 (11.6) |
| Neutral | 55 (40.1) | 85 (58.6) | 68 (48.6) | 102 (65.8) |
| Agree | 55 (40.1) | 33 (22.8) | 54 (38.6) | 26 (16.8) |
| Strongly agree | 0 | 4 (2.8) | 3 (2.1) | 6 (3.9) |
| All in all, you are inclined to feel you are a failure with drinkers |  |  |  |  |
| Strongly disagree | 4 (2.9) | 8 (5.5) | 2 (1.4) | 8 (5.2) |
| Disagree | 55 (40.1) | 52 (35.9) | 37 (26.4) | 33 (21.3) |
| Neutral | 65 (47.4) | 66 (45.5) | 75 (53.6) | 92 (59.4) |
| Agree | 13 (9.5) | 17 (11.7) | 26 (18.6) | 18 (11.6) |
| Strongly agree | 0 | 2 (1.4) | 0 | 4 (2.6) |
| Pessimism is the most realistic attitude to take towards drinkers |  |  |  |  |
| Strongly disagree | 3 (2.2) | 12 (8.3) | 5 (3.6) | 3 (1.9) |
| Disagree | 49 (35.8) | 48 (33.1) | 18 (12.9) | 26 (16.8) |
| Neutral | 59 (43.1) | 60 (41.4) | 90 (64.3) | 96 (61.9) |
| Agree | 23 (16.8) | 23 (15.9) | 26 (18.6) | 26 (16.8) |
| Strongly agree | 3 (2.2) | 2 (1.4) | 1 (0.7) | 4 (2.6) |
| Self-efficacy Scale |  |  |  |  |
| Raising the issue of alcohol with patients |  |  |  |  |
| Strongly disagree | 6 (4.4) | 14 (9.7) | 13 (9.3) | 7 (4.5) |
| Disagree | 69 (50.4) | 68 (46.9) | 46 (32.9) | 26 (16.8) |
| Neutral | 46 (33.6) | 42 (29.0) | 62 (44.3) | 79 (51.0) |
| Agree | 16 (11.7) | 19 (13.1) | 19 (13.6) | 39 (25.2) |
| Strongly agree | 0 | 2 (1.4) | 0 | 4 (2.6) |
| Using a screening test to explore current alcohol use of patients |  |  |  |  |
| Strongly disagree | 0 | 4 (2.8) | 4 (2.9) | 10 (6.5) |
| Disagree | 29 (21.2) | 25 (17.2) | 35 (25.0) | 29 (18.7) |
| Neutral | 49 (35.8) | 64 (44.1) | 65 (46.4) | 66 (42.6) |
| Agree | 59 (43.1) | 39 (26.9) | 35 (25.0) | 41 (26.5) |
| Strongly agree | 0 | 13 (9.0) | 1 (0.7) | 9 (5.8) |
| Explaining risks to health from different levels of alcohol consumption |  |  |  |  |
| Strongly disagree | 4 (2.9) | 8 (5.5) | 7 (5.0) | 3 (1.9) |
| Disagree | 65 (47.4) | 62 (42.8) | 42 (30.0) | 39 (25.2) |
| Neutral | 39 (28.5) | 52 (35.9) | 62 (44.3) | 72 (46.5) |
| Agree | 29 (21.2) | 21 (14.5) | 28 (20.0) | 35 (22.6) |
| Strongly agree | 0 | 2 (1.4) | 1 (0.7) | 6 (3.9) |
| Providing patients with ideas and practical advice on how to cut down |  |  |  |  |
| Strongly disagree | 0 | 1 (0.7) | 4 (2.9) | 3 (1.9) |
| Disagree | 43 (31.4) | 48 (33.1) | 32 (22.9) | 22 (14.2) |
| Neutral | 39 (28.5) | 48 (33.1) | 58 (41.4) | 76 (49.0) |
| Agree | 52 (38.0) | 42 (29.0) | 42 (30.0) | 48 (31.0) |
| Strongly agree | 3 (2.2) | 6 (4.1) | 4 (2.9) | 6 (3.9) |
| Helping patients to manage high risk drinking situations |  |  |  |  |
| Strongly disagree | 1 (0.7) | 0 | 4 (2.9) | 0 |
| Disagree | 29 (21.2) | 33 (22.8) | 26 (18.6) | 28 (18.1) |
| Neutral | 29 (21.2) | 42 (29.0) | 61 (43.6) | 76 (49.0) |
| Agree | 75 (54.7) | 58 (40.0) | 46 (32.9) | 42 (27.1) |
| Strongly agree | 3 (2.2) | 12 (8.3) | 3 (2.1) | 9 (5.8) |
| Avoiding blame and judgement when giving alcohol related advice to patients |  |  |  |  |
| Strongly disagree | 0 | 1 (0.7) | 10 (7.1) | 11 (7.1) |
| Disagree | 62 (45.3) | 75 (51.7) | 31 (22.1) | 33 (21.3) |
| Neutral | 62 (45.3) | 54 (37.2) | 75 (53.6) | 79 (51.0) |
| Agree | 13 (9.5) | 13 (9.0) | 24 (17.1) | 28 (18.1) |
| Strongly agree | 0 | 2 (1.4) | 0 | 4 (2.6) |
| Process |  |  |  |  |
| Planning Scale |  |  |  |  |
| The clinic provides clear goals for implementing SBI |  |  |  |  |
| Strongly disagree | 20 (14.6) | 10 (6.9) | 14 (10.0) | 18 (11.6) |
| Disagree | 42 (30.7) | 52 (35.9) | 24 (17.1) | 27 (17.4) |
| Neutral | 65 (47.4) | 64 (44.1) | 61 (43.6) | 60 (38.7) |
| Agree | 7 (5.1) | 17 (11.7) | 41 (29.3) | 48 (31.0) |
| Strongly agree | 3 (2.2) | 2 (1.4) | 0 | 2 (1.3) |
| Staff have clearly defined roles and responsibilities for implementing SBI |  |  |  |  |
| Strongly disagree | 20 (14.6) | 16 (11.0) | 6 (4.3) | 10 (6.5) |
| Disagree | 51 (37.2) | 54 (37.2) | 29 (20.7) | 35 (22.6) |
| Neutral | 46 (33.6) | 57 (39.3) | 62 (44.3) | 65 (41.9) |
| Agree | 20 (14.6) | 14 (9.7) | 43 (30.7) | 43 (27.7) |
| Strongly agree | 0 | 4 (2.8) | 0 | 2 (1.3) |
| Engaging |  |  |  |  |
| Some of our staff (i.e., managers, supervisors, other staff), have become program champions, actively supporting and promoting SBI beyond what is required |  |  |  |  |
| Strongly disagree | 35 (25.5) | 32 (22.1) | 33 (23.6) | 35 (22.6) |
| Disagree | 25 (18.2) | 22 (15.2) | 27 (19.3) | 33 (21.3) |
| Neutral | 22 (16.1) | 30 (20.7) | 27 (19.3) | 28 (18.1) |
| Agree | 48 (35.0) | 57 (39.3) | 44 (31.4) | 52 (33.5) |
| Strongly agree | 7 (5.1) | 4 (2.8) | 9 (6.4) | 7 (4.5) |
| Clinic staff takes an active interest in programmatic-related problems and successes |  |  |  |  |
| Strongly disagree | 28 (20.4) | 35 (24.1) | 26 (18.6) | 37 (23.9) |
| Disagree | 28 (20.4) | 22 (15.2) | 25 (17.9) | 26 (16.8) |
| Neutral | 26 (19.0) | 27 (18.6) | 33 (23.6) | 40 (25.8) |
| Agree | 45 (32.8) | 55 (37.9) | 42 (30.0) | 50 (32.3) |
| Strongly agree | 10 (7.3) | 6 (4.1) | 14 (10.0) | 2 (1.3) |
| Executing |  |  |  |  |
| Our clinic consistently implements programs (e.g. SBI) that are aligned with our mission and strategic plan |  |  |  |  |
| Strongly disagree | 31 (22.6) | 29 (20.0) | 31 (22.1) | 33 (21.3) |
| Disagree | 35 (25.5) | 35 (24.1) | 30 (21.4) | 30 (19.4) |
| Neutral | 34 (24.8) | 29 (20.0) | 30 (21.4) | 45 (29.0) |
| Agree | 33 (24.1) | 48 (33.1) | 45 (32.1) | 45 (29.0) |
| Strongly agree | 4 (2.9) | 4 (2.8) | 4 (2.9) | 2 (1.3) |
| Reflecting and Evaluating Scale |  |  |  |  |
| Throughout the clinic there is frequent and good communication about how different changes are going |  |  |  |  |
| Strongly disagree | 30 (21.9) | 35 (24.1) | 30 (21.4) | 32 (20.6) |
| Disagree | 21 (15.3) | 28 (19.3) | 30 (21.4) | 34 (21.9) |
| Neutral | 31 (22.6) | 35 (24.1) | 32 (22.9) | 31 (20.0) |
| Agree | 45 (32.8) | 43 (29.7) | 41 (29.3) | 50 (32.3) |
| Strongly agree | 10 (7.3) | 4 (2.8) | 7 (5.0) | 8 (5.2) |
| We use data to guide our clinic to implement SBI (e.g., performance reviews, assessments) |  |  |  |  |
| Strongly disagree | 25 (18.2) | 30 (20.7) | 22 (15.7) | 32 (20.6) |
| Disagree | 31 (22.6) | 27 (18.6) | 28 (20.0) | 31 (20.0) |
| Neutral | 30 (21.9) | 31 (21.4) | 39 (27.9) | 43 (27.7) |
| Agree | 45 (32.8) | 50 (34.5) | 41 (29.3) | 45 (29.0) |
| Strongly agree | 6 (4.4) | 7 (4.8) | 10 (7.1) | 4 (2.6) |
| Cultural attitudes toward drinking |  |  |  |  |
| Social approval of drinking |  |  |  |  |
| Drinking is considered a socially accepted practice in Hong Kong |  |  |  |  |
| Strongly disagree | 0 | 0 | 1 (0.7) | 3 (1.9) |
| Disagree | 3 (2.2) | 8 (5.5) | 5 (3.6) | 15 (9.7) |
| Neutral | 26 (19.0) | 19 (13.1) | 45 (32.1) | 48 (31.0) |
| Agree | 88 (64.2) | 112 (77.2) | 74 (52.9) | 74 (47.7) |
| Strongly agree | 20 (14.6) | 6 (4.1) | 15 (10.7) | 15 (9.7) |
| Functions of drinking in the Chinese Culture Scale |  |  |  |  |
| Drinking plays an important part in promoting interpersonal relations in Hong Kong |  |  |  |  |
| Strongly disagree | 0 | 0 | 0 | 1 (0.6) |
| Disagree | 13 (9.5) | 13 (9.3) | 12 (8.6) | 11 (7.1) |
| Neutral | 33 (24.1) | 41 (28.3) | 64 (45.7) | 76 (49.0) |
| Agree | 88 (64.2) | 89 (61.4) | 58 (41.4) | 61 (39.4) |
| Strongly agree | 3 (2.2) | 2 (1.4) | 6 (4.3) | 6 (3.9) |
| Alcohol is often used as a means of facilitating communication among people in Hong Kong |  |  |  |  |
| Strongly disagree | 0 | 1 (0.7) | 1 (0.7) | 0 |
| Disagree | 13 (9.5) | 10 (6.9) | 11 (7.9) | 9 (5.8) |
| Neutral | 36 (26.3) | 35 (24.1) | 60 (42.9) | 61 (39.4) |
| Agree | 85 (62.0) | 93 (64.1) | 64 (45.7) | 79 (51.0) |
| Strongly agree | 3 (2.2) | 6 (4.1) | 4 (2.9) | 6 (3.9) |
| Drinking with others is more encouraged than drinking alone in Hong Kong |  |  |  |  |
| Strongly disagree | 0 | 0 | 3 (2.1) | 0 |
| Disagree | 13 (9.5) | 5 (3.4) | 15 (10.7) | 10 (6.5) |
| Neutral | 29 (21.2) | 45 (31.0) | 51 (36.4) | 53 (34.2) |
| Agree | 82 (59.9) | 89 (61.4) | 68 (48.6) | 83 (53.5) |
| Strongly agree | 13 (9.5) | 6 (4.1) | 3 (2.1) | 9 (5.8) |
| People who host parties or social events tend to feel obliged to serve alcohol in Hong Kong |  |  |  |  |
| Strongly disagree | 0 | 0 | 1 (0.7) | 0 |
| Disagree | 6 (4.4) | 11 (7.6) | 6 (4.3) | 4 (2.6) |
| Neutral | 33 (24.1) | 31 (21.4) | 60 (42.9) | 68 (43.9) |
| Agree | 82 (59.9) | 87 (60.0) | 69 (49.3) | 65 (41.9) |
| Strongly agree | 16 (11.7) | 16 (11.0) | 4 (2.9) | 18 (11.6) |
| Serving alcohol or buying drinks is considered as part of treating people in Hong Kong |  |  |  |  |
| Strongly disagree | 0 | 0 | 1 (0.7) | 2 (1.3) |
| Disagree | 12 (8.8) | 11 (7.6) | 6 (4.3) | 2 (1.3) |
| Neutral | 23 (16.8) | 37 (25.5) | 58 (41.4) | 57 (36.8) |
| Agree | 95 (69.3) | 85 (58.6) | 71 (50.7) | 83 (53.5) |
| Strongly agree | 7 (5.1) | 12 (8.3) | 4 (2.9) | 11 (7.1) |
| Value of moderation proposed by Confucianism/ Taoism to control drinking |  |  |  |  |
| It is important that people should control the amount of alcohol they drink in any situation |  |  |  |  |
| Strongly disagree | 0 | 0 | 0 | 2 (1.3) |
| Disagree | 3 (2.2) | 7 (4.8) | 2 (1.4) | 2 (1.3) |
| Neutral | 20 (14.6) | 16 (11.0) | 53 (37.9) | 57 (36.8) |
| Agree | 65 (47.4) | 66 (45.5) | 64 (45.7) | 74 (47.8) |
| Strongly agree | 49 (35.8) | 56 (38.6) | 21 (15.0) | 20 (12.9) |
| Stigmatization issue of alcohol use |  |  |  |  |
| Addressing alcohol drinking problem may cause stigma-related issue of the patients in Hong Kong |  |  |  |  |
| Strongly disagree | 4 (2.9) | 3 (2.1) | 2 (1.4) | 0 |
| Disagree | 65 (47.4) | 39 (26.9) | 21 (15.0) | 22 (14.2) |
| Neutral | 55 (40.1) | 62 (42.8) | 69 (49.3) | 65 (41.9) |
| Agree | 10 (7.3) | 35 (24.1) | 44 (30.3) | 66 (42.6) |
| Strongly agree | 3 (2.2) | 6 (4.1) | 4 (2.9) | 2 (1.3) |

Supplementary table 3 Meaning of the scores of each factor

| Factors | A higher score of each factor means perceiving: |
| --- | --- |
| **Intervention characteristics** |  |
| Evidence Strength | a stronger evidence of supporting SBI |
| Relative advantage | more advantagous of using SBI compared to their prior approach |
| Adaptability Scale | better fit of SBI with the existing practice |
| Complexity | SBI to be more complex |
| Cost | SBI of higher cost |
| **Outer setting** |  |
| Cosmopolitanism | easier to refer patients with drinking problems |
| Patient Needs | better meet of patient needs |
| External policy or incentives | better external policy support |
| **Inner setting** |  |
| Relative priority | stronger need to implement SBI |
| Tension for change | greater importance to implement SBI |
| Network and communication | better network and communication among staff |
| Leadership engagement | better leadership supporting SBI implementation |
| Organizational Culture | better organizational culture supporting SBI implementation |
| Goals and Feedback | better goals and feedback on SBI implementation |
| Available Resources | more resources supporting SBI implementation |
| **Characteristics of individuals** |  |
| Knowledge | better knowledge of SBI |
| Positive Beliefs | more positive beliefs about SBI |
| Negative Beliefs | more negative beliefs about SBI |
| Self-efficacy | better self-efficacy to implement SBI |
| **Process** |  |
| Planning | better planning to implement SBI |
| Engaging | stronger involvement in SBI implementation among staff |
| Executing | better execution of programs in the clinic including SBI implementation |
| Reflecting and Evaluating | better evaluation of SBI implementation |
| Cultural attitudes toward drinking |  |
| Social approval of drinking | higher social approval of drinking |
| Functions of drinking in the  Chinese Culture Scale | more functions of drinking in the Chinese culture |
| Value of moderation proposed  by Confucianism/ Taoism to  control drinking | greater importance of not to drink to an excess proposed by Confucianism/Taoism |
| Stigmatization issue of alcohol use | higher stigmatization of alcohol use |

Supplementary table 4 Factors associated with the use of SBI (both AUDIT and BI) at least one episode in the past year (N=577)

|  | Physicians | | | | Nurses | | | |
| --- | --- | --- | --- | --- | --- | --- | --- | --- |
|  | Public (N=137) | | Private (N=145) | | Public (N=140) | | Private (N=155) | |
|  | OR (95%CI) | P values | OR (95%CI) | P values | OR (95%CI) | P values | OR (95%CI) | P values |
| Intervention characteristics |  |  |  |  |  |  |  |  |
| Evidence Strength Scale | 1.28  (1.03,1.60) | **0.03** | 1.34  (1.04,1.73) | **0.03** | 1.32  (1.07,1.62) | **0.01** | 1.25  (1.02,1.54) | **0.03** |
| Relative advantage | 1.09  (0.56,2.13) | 0.79 | 0.89  (0.50,1.59) | 0.70 | 0.91  (0.51,1.63) | 0.74 | 1.19  (0.68,2.08) | 0.55 |
| Adaptability Scale | 1.42  (1.22,1.67) | **0.02** | 1.24  (0.83,1.87) | 0.29 | 1.57  (1.14,2.17) | **0.007** | 1.07  (0.81,1.42) | 0.88 |
| Complexity Scale | 0.75  (0.58,0.96) | **0.02** | 0.78  (0.60,0.99) | **0.049** | 0.90  (0.77,1.06) | 0.21 | 0.83  (0.70,0.97) | **0.04** |
| Cost | 0.44  (0.25,0.79) | **0.005** | 0.46  (0.29,0.73) | **0.001** | 0.57  (0.34,0.94) | **0.03** | 0.68  (0.44,1.07) | 0.09 |
| Outer setting |  |  |  |  |  |  |  |  |
| Cosmopolitanism | 1.21  (0.94,2.26) | 0.08 | 1.04  (0.72,1.51) | 0.84 | 0.96  (0.69,1.34) | 0.83 | 1.07  (0.78,1.47) | 0.66 |
| Patient Needs Scale | 1.12  (0.92,1.37) | 0.26 | 1.01  (0.83,1.22) | 0.92 | 0.87  (0.72,1.05) | 0.16 | 1.03  (0.87,1.21) | 0.77 |
| External policy or incentives | 1.60  (0.93,2.75) | 0.09 | 0.83  (0.50,1.36) | 0.45 | 1.60  (0.99,2.77) | 0.05 | 1.09  (0.74,1.60) | 0.67 |
| Inner setting |  |  |  |  |  |  |  |  |
| Relative priority | 1.98  (1.19,3.27) | **0.008** | 2.01  (1.15,3.52) | **0.01** | 0.73  (0.50,1.09) | 0.12 | 1.06  (0.73,1.54) | 0.64 |
| Tension for change | 2.31  (1.26,4.26) | **0.007** | 1.94  (1.09,3.47) | **0.03** | 0.98  (0.84,1.15) | 0.80 | 1.44  (0.92,2.26) | 0.11 |
| Network and communication | 1.09  (0.59,2.03) | 0.78 | 0.79  (0.43,1.47) | 0.48 | 0.80  (0.49,1.31) | 0.38 | 1.00  (0.60,1.67) | 0.99 |
| Leadership engagement | 2.30  (1.30,4.07) | **0.004** | 0.82  (0.49,1.46) | 0.55 | 1.45  (0.95,2.21) | 0.09 | 1.08  (0.73,1.58) | 0.71 |
| Organizational Culture Scale | 1.05  (0.93,1.18) | 0.47 | 1.07  (0.93,1.23) | 0.35 | 1.17  (1.05,1.30) | **0.004** | 1.22  (0.98,1.71) | **<0.001** |
| Goals and Feedback Scale | 0.99  (0.73,1.35) | 0.97 | 0.93  (0.67,1.29) | 0.66 | 1.01  (0.79,1.29) | 0.95 | 0.83  (0.63,1.08) | 0.17 |
| Available Resources Scale | 1.24  (1.04,1.48) | **0.03** | 1.35  (1.10,1.75) | **0.009** | 1.27  (1.09,1.48) | **0.003** | 1.20  (1.09,1.33) | **0.009** |
| Characteristics of individuals |  |  |  |  |  |  |  |  |
| Knowledge Scale | 1.52  (1.07,2.16) | **0.02** | 1.45  (1.02,2.08) | **0.04** | 1.22  (1.04,1.43) | **0.01** | 1.22  (1.04,1.43) | **0.01** |
| Positive Beliefs Scale | 0.98  (0.77,1.24) | 0.86 | 0.80  (0.62,1.02) | 0.07 | 1.10  (0.89,1.36) | 0.37 | 1.10  (0.90,1.35) | 0.37 |
| Negative Beliefs Scale | 0.81  (0.63,1.05) | 0.11 | 0.90  (0.70,1.16) | 0.41 | 1.12  (0.88,1.43) | 0.37 | 0.82  (0.66,1.03) | 0.09 |
| Self-efficacy Scale | 1.15  (1.01,1.31) | **0.04** | 1.13  (0.98,1.27) | 0.09 | 1.13  (1.02,1.25) | **0.02** | 1.16  (1.04,1.29) | **0.01** |
| Process |  |  |  |  |  |  |  |  |
| Planning Scale | 1.36  (1.00,1.83) | **0.04** | 1.56  (1.12,2.17) | **0.009** | 1.35  (1.05,1.75) | **0.02** | 1.27  (1.02,1.60) | **0.04** |
| Engaging Scale | 0.83  (0.51,1.35) | 0.45 | 1.13  (0.70,1.80) | 0.62 | 0.67  (0.44,1.03) | 0.07 | 1.00  (0.65,1.52) | 0.99 |
| Executing | 0.72  (0.47,1.11) | 0.14 | 1.13  (0.77,1.65) | 0.54 | 1.26  (0.92,1.71) | 0.15 | 0.81  (0.59,1.10) | 0.17 |
| Reflecting and Evaluating Scale | 1.14  (0.83,1.56) | 0.41 | 0.95  (0.70,1.29) | 0.73 | 1.14  (0.89,1.47) | 0.29 | 1.00  (0.80,1.27) | 0.97 |
| Cultural attitudes toward drinking |  |  |  |  |  |  |  |  |
| Social approval of drinking | 0.41  (0.22,0.79) | **0.007** | 0.27  (0.13,0.54) | **<0.001** | 0.49  (0.31,0.77) | **0.002** | 0.59  (0.37,0.93) | **0.02** |
| Functions of drinking in the  Chinese Culture Scale | 1.09  (0.93,1.28) | 0.28 | 0.88  (0.75,1.03) | 0.12 | 1.01  (0.89,1.15) | 0.86 | 0.91  (0.79,1.05) | 0.22 |
| Value of moderation proposed  by Confucianism/ Taoism to  control drinking | 1.31  (0.84,2.05) | 0.07 | 1.26  (0.81,1.96) | 0.08 | 1.29  (0.76,2.28) | 0.08 | 1.32  (0.82,2.29) | 0.06 |
| Stigmatization issue of  alcohol use | 0.89  (0.53,1.64) | 0.13 | 0.91  (0.52,1.71) | 0.11 | 0.81  (0.55,1.41) | 0.12 | 0.84  (0.47,1.49) | 0.15 |

AUDIT: Alcohol Use Disorders Identification Test

BI: brief intervention

OR: odds ratio

CI: confidence interval
